# Starvation Exacerbates Cold-Induced Synergistic Hepatic Injury in *Pelteobagrus vachelli* via Gut–Liver Axis Disruption and Ferroptosis-Related Metabolic Reprogramming

**DOI:** 10.3390/antiox15080962

**Published:** 2026-07-31

**Authors:** Amei Liu, Libo Yang, Yuting Hu, Huaxing Zhou, Huan Wang, Guoqing Duan

**Affiliations:** 1 Institute of Fisheries Science, Anhui Academy of Agricultural Sciences, Nongke South Road, Hefei 230031, China; 2 Anhui Province Key Laboratory of Aquaculture & Stock Enhancement, Hefei 230031, China; 3 Anhui Agricultural Germplasm Resource Center, Anhui Academy of Agricultural Sciences, Hefei 230031, China; 4 Institute of Artificial Intelligence, Hefei Comprehensive National Science Center, Hefei 230088, China

**Keywords:** *Pelteobagrus vachelli*, overwintering, combined stress, metabolic reprogramming, gut microbiota, oxidative stress

## Abstract

Overwintering represents a critical bottleneck for farmed fish, during which low temperature and starvation stress frequently co-occur, yet their synergistic effects on fish health remain poorly understood. Here, we exposed *Pelteobagrus vachelli*, a cold-sensitive freshwater species, to four conditions for 10 days: control (25 °C, feeding), starvation alone (25 °C, starvation), cold alone (11 °C, feeding), and combined cold–starvation (11 °C, starvation). Hepatic histopathology, antioxidant and liver function indices, gut microbiota (16S rRNA sequencing), and untargeted metabolomics (LC–MS) were integrated to elucidate gut–liver axis mechanisms underlying synergistic injury. Combined stress synergistically aggravated liver injury, as evidenced by hepatocellular vacuolation and necrosis, elevated aspartate aminotransferase (AST), alanine transaminase (ALT), malondialdehyde (MDA), and suppressed total superoxide dismutase (T–SOD), glutathione (GSH), catalase (CAT), and total antioxidant capacity (T–AOC). Two-way ANOVA confirmed significant interactive effects between cold and starvation stress (*p* < 0.05). Multi-omics revealed that dual stress uniquely activated multiple cell death pathways (FoxO, autophagy, ferroptosis, apoptosis), with marked oxidative phosphorylation (OXPHOS) activation and glutathione depletion—a signature absent under single stressors. Gut microbiota restructuring showed beneficial commensals (*Cetobacterium*, *Prevotella*, *Lactobacillus*) depleted and opportunistic pathogens (*Plesiomonas*, *Pseudomonas*, *Flavobacterium*) enriched, with *Plesiomonas* identified as the dominant biomarker (LDA score = 5.32). Integrative networks identified these pathogenic genera as hubs linking metabolic dysregulation to liver damage. Collectively, combined cold–starvation induces synergistic liver injury via gut–liver axis disruption, driving metabolic reprogramming and oxidative damage. These findings provide candidate biomarkers for overwintering stress monitoring and inform management strategies to mitigate cold–starvation-induced hepatic injury in aquaculture.

## 1. Introduction

Global climate change has intensified seasonal temperature fluctuations and extreme weather events, posing severe threats to the stability of aquatic ecosystems and the sustainable development of aquaculture [1,2]. In temperate and subtropical aquaculture regions, farmed fish experience combined environmental stressors during overwintering: natural water temperature drops drastically, reducing food availability, while periodic feed withdrawal in aquaculture operations further exacerbates nutritional deficiency. These two stressors frequently co-occur and constitute unavoidable challenges to fish health [3,4]. Such combined exposure readily disrupts physiological homeostasis, compromises stress resistance, and can trigger mass mortality, representing a primary driver of overwintering production losses in aquaculture [3,5]. Although the physiological responses of fish to single low-temperature or starvation stress have been extensively studied, systematic evidence remains scarce regarding whether concurrent exposure to both stressors induces non-additive synergistic injuries distinct from the simple summation of individual effects, and what regulatory mechanisms underlie such responses.

Existing evidence demonstrates that single low-temperature stress elicits multi-dimensional impairments in fish by disrupting intestinal microbiota homeostasis, inhibiting feeding and metabolism, and impairing antioxidant-immune balance [6,7,8,9,10,11,12]. For instance, low temperature disturbs hepatic antioxidant systems in black porgy (*Acanthopagrus schlegelii*) and Atlantic salmon (*Salmo salar*) [8,13,14], and triggers hepatocellular lipid peroxidation and apoptosis in freshwater drum (*Aplodinotus grunniens*) [15]. In contrast, single starvation primarily drives adaptive responses to nutrient deprivation through the mobilization of energy reserves, activation of autophagic pathways, and global metabolic reprogramming [16,17,18]. Additionally, starvation markedly remodels intestinal microbial composition and structure, as documented in rainbow trout (*Oncorhynchus mykiss*), grass carp (*Ctenopharyngodon idella*), and largemouth bass (*Micropterus salmoides*) [19,20,21,22]. Recent studies across multiple teleost species have further indicated that the biological impacts of co-occurring cold and food limitation cannot be adequately described as a linear superposition of responses observed under each stressor alone [3,23].

The liver, as the central metabolic organ governing energy homeostasis, detoxification, and immune defense in fish, plays an indispensable role in environmental stress adaptation [7,11,24]. Single cold stress induces hepatocellular vacuolar degeneration, glycogen depletion, and aberrant lipid accumulation—pathological hallmarks of oxidative injury and redox imbalance [7,8,13,14], whereas single starvation remodels hepatic metabolism by activating fatty acid oxidation and ketogenesis, frequently accompanied by compromised antioxidant defense and enhanced lipid peroxidation [25,26,27]. Nevertheless, whether the liver exhibits unique metabolic reprogramming signatures that exceed the phenotypic alterations induced by either single stressor upon concurrent exposure remains unclear.

The gut–liver axis serves as a core bidirectional signaling hub connecting intestinal microecology, intestinal barrier integrity, and hepatic function, and plays a pivotal role in fish responses to environmental stress [24,28]. Numerous studies have confirmed that disruption of gut–liver axis homeostasis is a major driver of hepatic damage, with impaired intestinal morphology and dysbiosis serving as primary triggers of gut–liver communication breakdown [29,30,31]. Under single stress conditions, low temperature increases intestinal permeability and reshapes microbial composition [32,33], while starvation deteriorates intestinal histology and alters gut microbial diversity [22,34]. However, most prior investigations have focused on individual stressors, leaving the regulatory mechanisms under combined low-temperature and starvation stress largely unexplored. It remains to be clarified whether co-occurring cold and starvation exacerbate hepatic metabolic disorders and amplify oxidative damage through the disruption of redox homeostasis via the interactive ‘microbiota–metabolites–physiological indicators’ network, driven by depletion of beneficial microbes and enrichment of opportunistic pathogens in the intestine, and whether such interactive network involves novel regulatory modules absent under single stress conditions.

*Pelteobagrus vachelli* is an economically important freshwater aquaculture species in China, characterized by large-scale cultivation and high commercial value. However, its extreme sensitivity to low temperature, coupled with high overwintering mortality, severely constrains the sustainable development of its breeding industry [23]. Our previous work has characterized the hepatic physiological and intestinal microbial responses of *P. vachelli* under single low-temperature stress [35]. Nevertheless, under natural overwintering scenarios, it remains unknown whether starvation further attenuates endogenous compensatory protective capacity and exacerbates cold-induced hepatic damage, and whether the gut–liver axis mediates the synergistic regulation linking intestinal microbiota and hepatic metabolism. Accordingly, we propose the central hypothesis of this study: combined low-temperature and starvation stress exerts synergistic hepatotoxic effects in overwintering *P. vachelli*, with injury severity significantly exceeding the additive effects of individual stressors. Such synergistic damage is mediated by the disruption of gut–liver axis homeostasis, accompanied by a distinctive metabolic shift characterized by glutathione depletion, oxidative phosphorylation dysregulation, and ferroptosis—a recently recognized oxidative-stress-driven cell death pathway—distinct from those activated by single stressors.

To verify this hypothesis, four experimental groups were established: control (25 °C, feeding), single starvation (25 °C, fasting), single cold (11 °C, feeding), and combined cold–starvation (11 °C, fasting). Hepatic histopathology and biochemistry, 16S rRNA gut microbiota sequencing, and LC–MS untargeted metabolomics were integrated to systematically characterize the synergistic injury mechanism. Multi-omics correlation networks were constructed to identify interactive modules comprising core microbial taxa, characteristic metabolites, and key biochemical indicators, with the ultimate goal of elucidating the intrinsic mechanism by which co-occurring low temperature and starvation induce synergistic hepatic damage via gut–liver axis dysfunction. The findings of this study will enrich the theoretical framework for understanding fish physiological responses to combined overwintering stress, and provide scientific support for stress-resistant aquaculture strategies and overwinter health management.

## 2. Materials and Methods

### 2.1. Fish and Experimental Design

A total of 360 healthy *P. vachelli* (mean body weight: 35.32 ± 2.56 g; body length: 14.56 ± 0.68 cm) were obtained from the Fisheries Research Institute of Anhui Academy of Agricultural Sciences. All fish were reared in a recirculating aquaculture system and acclimated for two weeks. During acclimation, water temperature was maintained at 25 ± 1 °C, dissolved oxygen at 6.5 ± 0.5 mg/L, and pH at 7.1 ± 0.5. Fish were fed commercial feed twice daily at 2% of their body weight (*w*/*w*).

**Figure 1:**
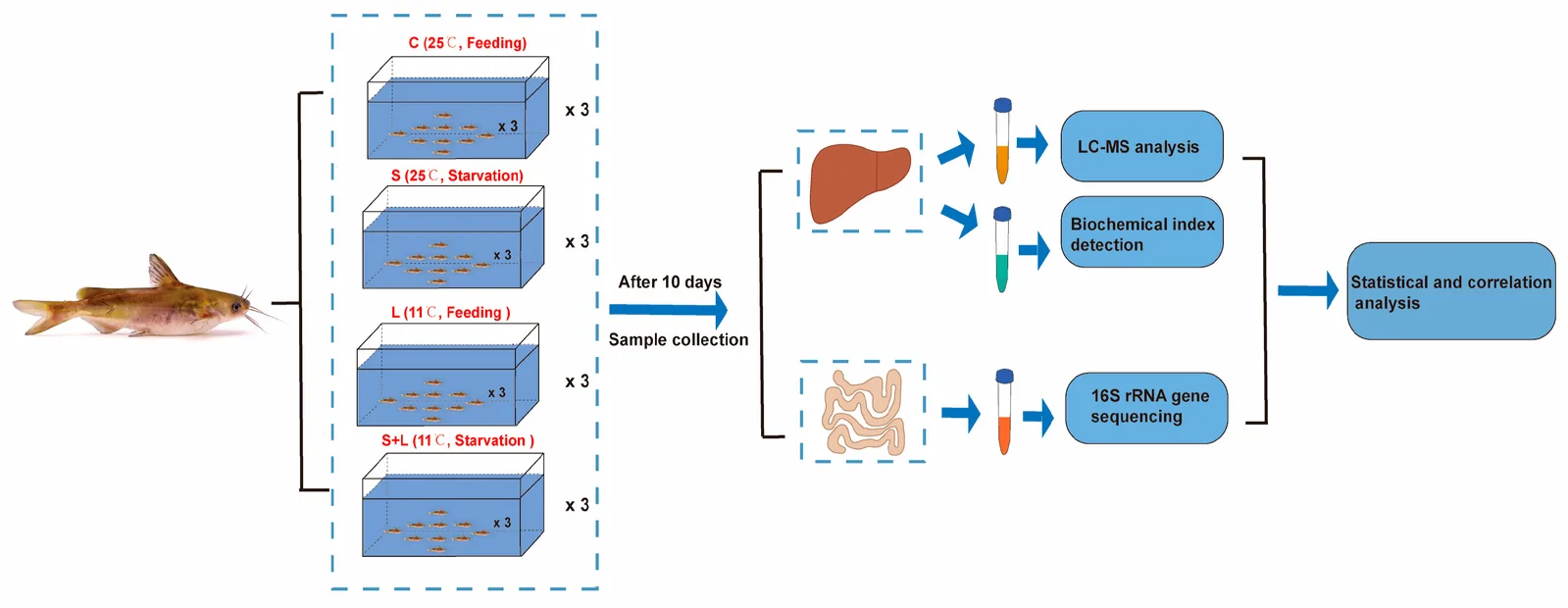
Scheme of the experimental design.

After acclimation, fish were randomly assigned to four experimental groups: control (C, 25 °C, feeding), starvation alone (S, 25 °C, starvation), cold stress alone (L, 11 °C, feeding), and combined cold and starvation stress (S + L, 11 °C, starvation). Each group comprised three replicate tanks (*n* = 3 per group), with 30 fish per tank. For analytical measurements, nine fish were randomly selected from each tank and pooled into a single composite sample, with each tank processed as one biological replicate (Figure 1). The water temperature in the cold exposed groups (L and S + L) was gradually reduced from 25 °C to 11 °C at a rate of 0.5 °C/h. The experiment lasted for 10 days. During the experimental period, water quality was maintained by daily siphoning of uneaten feed and feces.

After two weeks of acclimation, *P. vachelli* were randomly assigned to four groups: control (C, 25 °C, feeding), starvation (S, 25 °C, starvation), cold stress (L, 11 °C, feeding), and combined cold and starvation stress (S + L, 11 °C, starvation). Each group had three biological replicate tanks. After 10 days of treatment, liver and intestinal samples were collected for LC-MS metabolomics, biochemical measurement, 16S rRNA sequencing, and integrative correlation analyses.

### 2.2. Sample Collection

At the end of the 10-day experiment, fish were randomly selected from each tank and anesthetized with 0.02% tricaine methanesulfonate (MS–222). Liver tissues obtained from the nine fish within each tank were pooled into one composite sample and aliquoted for biochemical assays and metabolomic analysis. Intestinal contents were collected from the same individuals pooled per tank for gut microbiota analysis. All samples were immediately snap frozen in liquid nitrogen and stored at −80 °C until further processing (Figure 1). Additionally, liver tissues from three randomly selected fish per tank were fixed in 4% paraformaldehyde for histological examination.

### 2.3. Histological Analysis

Liver tissue samples were fixed in 4% paraformaldehyde for 48 h, dehydrated through a graded ethanol series (50%, 75%, 95%, and absolute ethanol), cleared in xylene, and embedded in paraffin. Sections (5 µm) were cut and stained with hematoxylin and eosin (H&E) [36]. Stained sections were observed and photographed under an optical microscope (Olympus, Tokyo, Japan). Quantification of nuclear area and vacuolation was performed using ImageJ software (NIH ImageJ, Version 2, Bethesda, MD, USA) by measuring pixel intensity and the vacuole to cytoplasm ratio in 10 randomly selected cells per tissue section (*n* = 3 sections per sample). A total of 30 cells per treatment group were analyzed.

### 2.4. Determination of Antioxidant Enzyme Activities and Hepatic Injury Indices

Liver tissues were homogenized in ice-cold physiological saline (0.9% NaCl) at a ratio of 1: 9 (*w*/*v*) to prepare a 10% homogenate. The homogenate was centrifuged at 3500× *g* for 10 min at 4 °C, and the supernatant was collected for biochemical analysis. Activities of total superoxide dismutase (T–SOD, A001-3-2), reduced glutathione (GSH, A006-2-1), aspartate aminotransferase (AST, C010-2-1), alanine aminotransferase (ALT, C009-3-2), catalase (CAT, A007-2-1), and malondialdehyde (MDA, A003-4-1) content were determined using commercial kits (Nanjing Jiancheng Bioengineering Institute, Nanjing, China) according to the manufacturer’s instructions. Total protein concentration was measured by the Coomassie Brilliant Blue method prior to activity calculations. Each treatment group had three biological replicates.

### 2.5. Gut Microbiota Analysis

#### 2.5.1. DNA Extraction and 16S rRNA Gene Sequencing

Microbial genomic DNA was extracted from intestinal contents using the E.Z.N.A.^®^ Mag–Bind^®^ Stool DNA Kit (Omega Bio tek, Norcross, GA, USA). The V3–V4 hypervariable region of the bacterial 16S rRNA gene was amplified by PCR using previously described primers and cycling conditions [37]. Purified amplicons were normalized to equal molar concentrations and subjected to paired-end 150 (PE150) sequencing on an Illumina NovaSeq 6000 platform (Illumina Inc., San Diego, CA, USA).

#### 2.5.2. Bioinformatic Analysis

Microbial community composition was analyzed at phylum and genus levels. Alpha diversity was estimated using the Chao1 and Shannon indices, and beta diversity was assessed by principal coordinate analysis (PCoA) based on Bray–Curtis distances, with PERMANOVA (999 permutations) used to test significant differences among groups. Linear discriminant analysis effect size (LEfSe) was performed via the Galaxy web platform (https://huttenhower.sph.harvard.edu/galaxy/, accessed on 19 March 2025) with an LDA score > 4.0.

#### 2.5.3. Functional Prediction of Gut Microbiota

Functional capabilities of the gut microbiota were predicted using PICRUSt2 (Phylogenetic Investigation of Communities by Reconstruction of Unobserved States) based on the KEGG (Kyoto Encyclopedia of Genes and Genomes) database. It should be emphasized that these functional profiles are computationally predicted outcomes, rather than experimentally measured functional data. Predicted functional pathways at KEGG levels 2 and 3 were compared among the four experimental groups.

### 2.6. Metabolomic Analysis

#### 2.6.1. Untargeted LC MS Metabolomics

Liver samples were subjected to untargeted metabolomic analysis using an ultra-high performance liquid chromatography system (1290 Infinity LC, Agilent Technologies, Santa Clara, CA, USA) coupled with a quadrupole time of flight mass spectrometer (QTOF/MS 6545, Agilent Technologies). Chromatographic separation was performed on an ACQUITY UPLC HSS T3 column (100 mm × 2.1 mm, 1.8 μm) with mobile phase A (water containing 0.1% formic acid) and mobile phase B (acetonitrile) under a gradient elution program. Mass spectrometry detection was achieved using a heated electrospray ionization (HESI) source operating in both positive and negative ion modes. Metabolite extraction and LC-MS parameters were performed as previously described [38].

#### 2.6.2. Differential Metabolite Identification

LC-MS data were processed for peak detection, alignment, and normalization. Multivariate statistical analysis was carried out using SIMCA software (version 14.1, Umetrics, MKS Data Analytics Solutions, Umea, Sweden). Orthogonal partial least squares discriminant analysis (OPLS DA) was employed to visualize group separation. Differentially expressed metabolites (DEMs) were identified based on variable importance in projection (VIP) scores ≥ 1.0. Volcano plots were generated to visualize the distribution of DEMs.

#### 2.6.3. Metabolic Pathway Enrichment Analysis

KEGG pathway enrichment analysis of DEMs was performed using the MetaboAnalyst 5.0 web platform (http://www.metaboanalyst.ca, accessed on 15 October 2024). Significantly enriched pathways were defined by a hypergeometric test with *p* value < 0.05.

### 2.7. Correlation and Integrative Network Analysis

#### 2.7.1. Correlation Analysis

Spearman’s rank correlation analysis was performed to evaluate associations between (i) differentially abundant gut bacterial genera and hepatic biochemical indicators, and (ii) differential bacterial genera and DEMs. Correlation matrices were visualized as hierarchical clustering heatmaps generated with the pheatmap package (v1.0.12) in R (R Core Team, 2023). Statistical significance was determined using the Benjamini–Hochberg false discovery rate (FDR) correction at a threshold of q < 0.05.

#### 2.7.2. Multi-Omics Integrative Network Analysis

A multi-omics correlation network was constructed to integrate gut microbiota, hepatic metabolome and biochemical phenotypes based on all significant pairwise Spearman correlations (q < 0.05). Network visualization was completed in Cytoscape v3.9.1 (https://cytoscape.org/, accessed on 21 April 2026).

### 2.8. Statistical Analysis

All data are presented as mean ± SEM. GraphPad Prism 9.0 (GraphPad Software, San Diego, CA, USA) was used for statistical calculations and figure plotting. Two-way ANOVA followed by Tukey‘s post hoc test was used to evaluate the interactive effects of temperature and feeding status on all measured parameters. PERMANOVA for gut microbiota beta diversity was performed with 999 permutations. LEfSe was performed with an LDA threshold > 4. Spearman’s correlation analysis was performed with Benjamini–Hochberg FDR correction (q < 0.05). Significance was accepted at *p* < 0.05. All analyses were based on *n* = 3 biological replicates per group as described in Section 2.1.

## 3. Results

### 3.1. Histopathological Alterations of the Liver Under Cold and Starvation Stress

**Figure 2:**
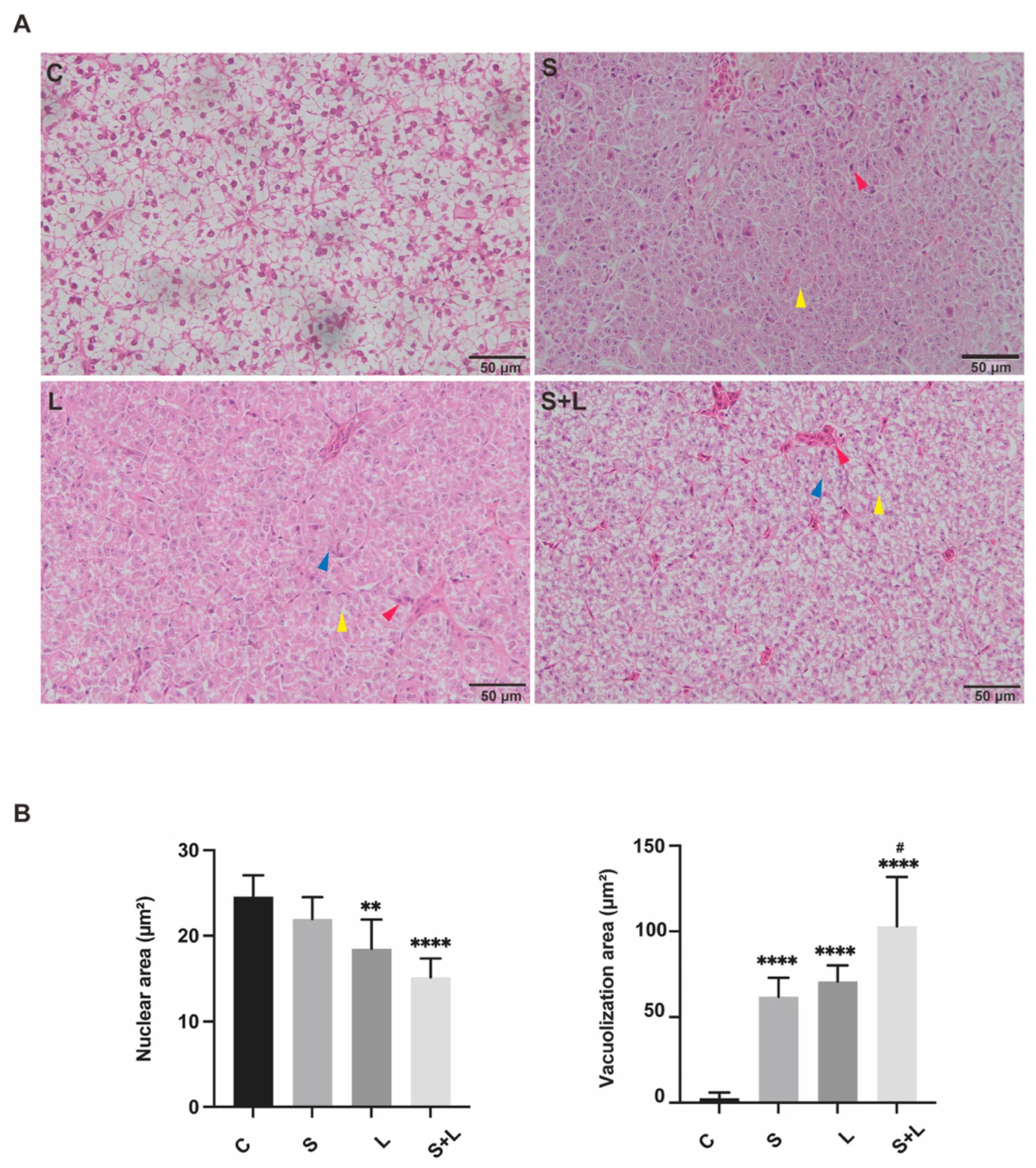
Hepatic histopathology of *P. vachelli* under cold and starvation stress. (**A**) Representative hematoxylin and eosin (H&E)-stained liver sections. Red arrowheads indicate focal inflammatory cell infiltration. Yellow arrowheads mark hepatocytes exhibiting characteristic ballooning degeneration with extensive cytoplasmic vacuolization, ill-defined borders, and loosely arranged, reticular cytoplasm. Blue arrowheads denote nuclear abnormalities, including pyknosis (condensation) and karyolysis (nuclear disappearance), which are hallmarks of programmed cell death. The control (C), starvation alone (S), cold alone (L), and combined (S + L) group. (**B**) Quantitative analysis of liver vacuolation area and nuclear morphological changes. All data are expressed as mean ± standard error of the mean (SEM), with *n* = 3 biological replicates per group. Asterisks (** *p* < 0.01; **** *p* < 0.0001) indicate statistically significant differences relative to the C group. The hash symbol (# *p* < 0.05) denotes a significant difference between the L and S + L treatment groups. Scale bars correspond to 50 μm.

Histopathological alterations in the liver of *P. vachelli* were assessed following a 10-day exposure to starvation, cold stress, or their combination (Figure 2A). H&E staining revealed intact hepatic architecture in the control group, characterized by densely packed, regularly arranged hepatocytes with homogeneous cytoplasm, and an absence of vacuolar degeneration or inflammatory infiltration.

In the starvation-only group, hepatocellular boundaries remained largely discernible. Only a few scattered inflammatory cells and mild vacuolization were observed, while nuclear morphology appeared essentially normal. The cold stress group exhibited more pronounced pathological changes, including blurred cell borders, nuclear pyknosis (reduced nuclear area), increased cytoplasmic vacuolation, and mild inflammatory infiltration. Relative to the cold stress group, the combined cold and starvation group displayed exacerbated hepatic injury. This group featured more extensive vacuolar degeneration, more widespread nuclear pyknosis, denser aggregation of inflammatory cells, and the emergence of focal tissue erosion—a lesion absent in the cold stress group. Moreover, hepatocyte organization was nearly completely disrupted. Quantitative morphometric analysis of nuclear area and vacuolation area further corroborated these histological observations and suggested a synergistic aggravating effect of the combined stress (Figure 2B).

### 3.2. Hepatic Antioxidant and Liver Function Responses Respond to Cold and Starvation Stress

**Figure 3:**
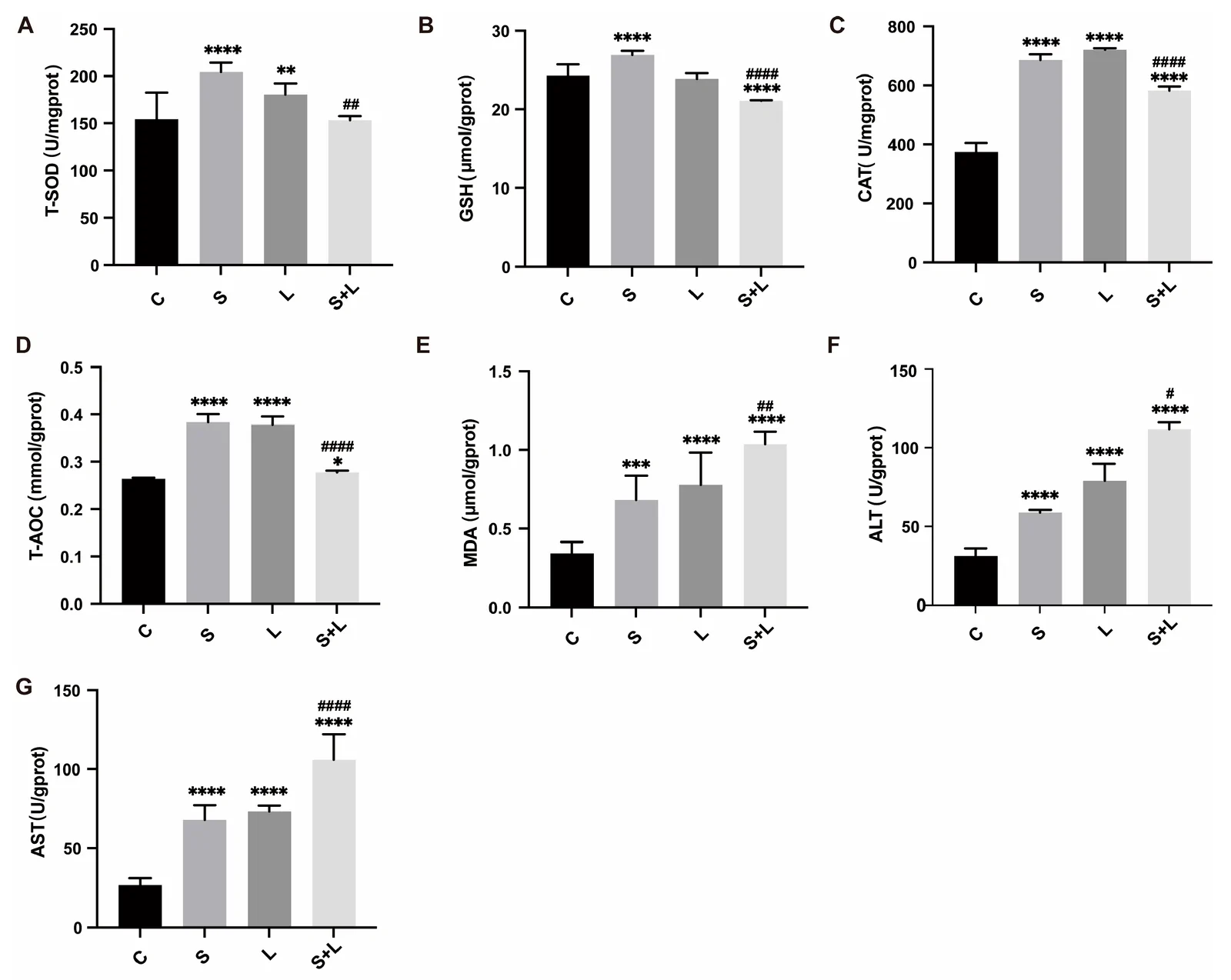
Effects of combined cold and starvation stress on hepatic biochemical parameters in *P. vachelli*. (**A**) Total superoxide dismutase (T-SOD), (**B**) reduced glutathione (GSH), (**C**) catalase (CAT) activities, (**D**) total antioxidant capacity (T-AOC), (**E**) malondialdehyde (MDA) content, (**F**) alanine aminotransferase (ALT), and (**G**) aspartate aminotransferase (AST) activities. Asterisks indicate significant differences compared with the control group (* *p* < 0.05, ** *p* < 0.01, *** *p* < 0.001, **** *p* < 0.0001). Hash symbols denote significant differences between the L and S + L groups (# *p* < 0.05, ## *p* < 0.01, #### *p* < 0.0001). Data are presented as means ± standard error of the mean (SEM), with *n* = 3 biological replicates per group (nine fish pooled per replicate). The control (C), starvation alone (S), cold alone (L), and combined (S + L) group.

Hepatic antioxidant capacity and liver function markers were evaluated in the control (C), starvation alone (S), cold alone (L), and combined stress (S + L) groups to comprehensively assess liver injury induced by combined cold and starvation stress (Figure 3). As shown in Figure 3A–D, activities of key antioxidant enzymes (T–SOD, GSH, CAT) and total antioxidant capacity (T–AOC) were significantly elevated in both S and L relative to the control group. Notably, although remaining above control levels, these parameters were markedly lower in the S + L than in either single-stress group. Conversely, MDA content, a classical marker of lipid peroxidation, showed a progressive increase across the four groups, peaking in the S + L group and significantly exceeding that in the L (Figure 3E). These findings suggest that single stress elicits a compensatory antioxidant response, whereas combined stress overwhelms this defense, leading to excessive oxidative stress in the liver.

Liver function biomarkers further corroborated the extent of hepatic injury. Compared with the control group, ALT and AST activities were significantly elevated in all stress-exposed groups, with the highest levels observed in the S + L group, which were significantly greater than those in either single-stress group (Figure 3F, G). Two-way ANOVA revealed a significant interactive effect between temperature and feeding status on both ALT and AST activities (*p* < 0.05). Simple effects analysis further demonstrated that under cold condition (11 °C), the S + L group exhibited significantly higher ALT and AST activities than the L group (*p* < 0.05), confirming that starvation exacerbates cold-induced hepatocellular injury (Figure S1). These changes indicate severe disruption of normal hepatic physiological function under dual stress, with the synergistic interaction between cold and starvation serving as a key driver of exacerbated liver damage.

### 3.3. Integrated Metabolomic Profiling Reveals Distinct Hepatic Metabolic Signatures Under Cold and Starvation Stress

**Figure 4:**
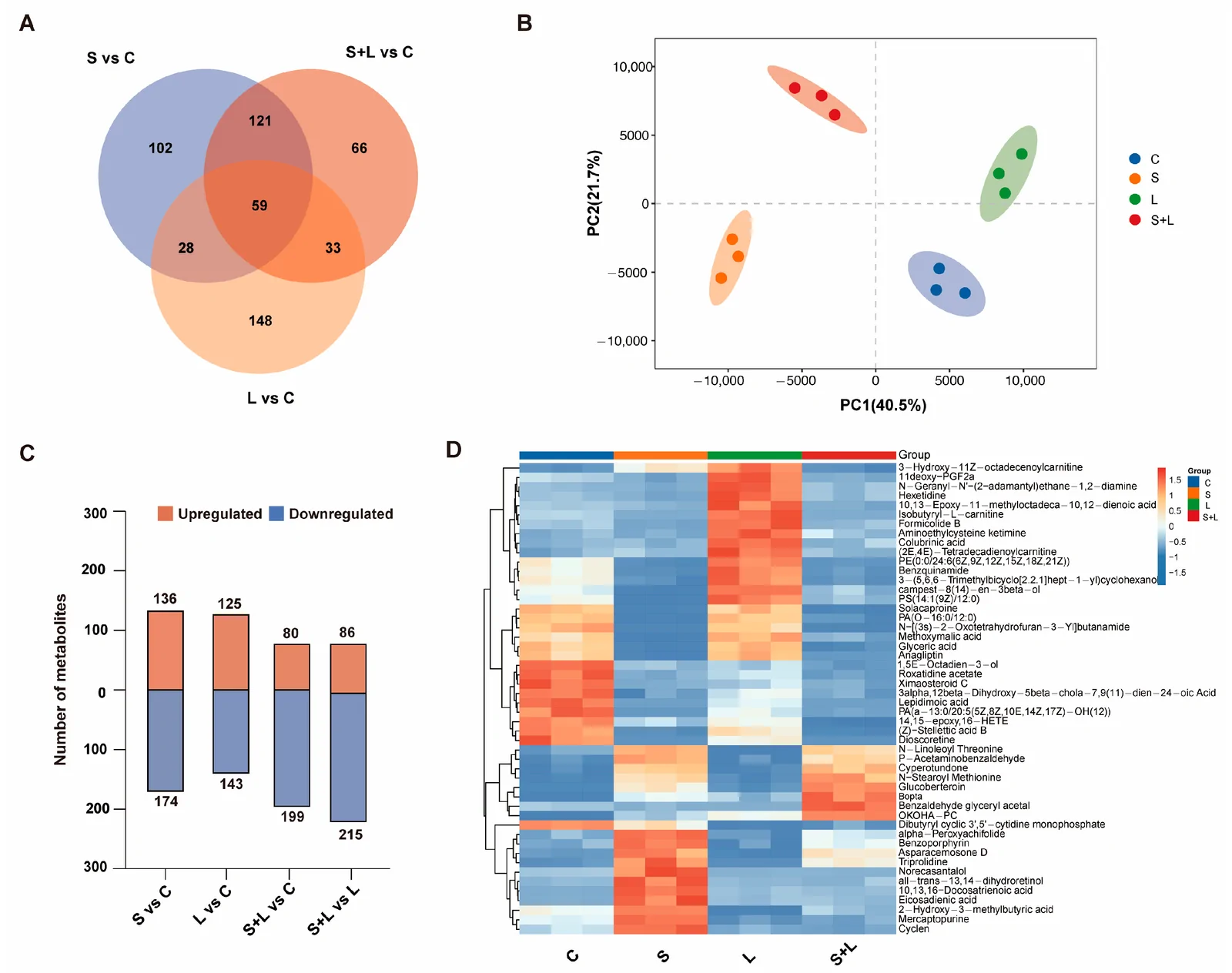
Comprehensive metabolomic profiling reveals the effects of cold stress and starvation on the liver of *P. vachelli*. (**A**) Venn diagram showing the overlap of differentially expressed metabolites (DEMs) across three comparative groups. (**B**) Principal component analysis (PCA) of all metabolites from nine individuals, depicting the variance distribution among samples. (**C**) Quantitative statistics of DEMs: bar graph illustrating the number of upregulated and downregulated metabolites between groups. (**D**) Heatmap displaying the expression profiles of top 50 DEMs in the liver. The control (C), starvation alone (S), cold alone (L), and combined (S + L) group.

To characterize hepatic metabolic responses to single and combined stress, untargeted metabolomics profiling was performed on liver samples from the C, S, L, and S + L groups. The Venn diagram (Figure 4A) shows the overlap of differentially expressed metabolites (DEMs) among the three pairwise comparisons versus the control (S vs. C, L vs. C, and S + L vs. C). A total of 59 DEMs were shared across all three stress conditions, indicating a common core metabolic response to stress. These shared DEMs represent metabolites significantly altered in all three comparisons regardless of direction (see Figure 4C for up/down distribution). Notably, the combined stress group (S + L vs. C) exhibited the highest number of unique DEMs (66), suggesting that dual stress induces a distinct metabolic signature. Principal component analysis (PCA) based on all detected metabolites revealed clear separation among the four groups (Figure 4B). The S + L group formed a distinct cluster separate from both single-stress groups and the control, demonstrating that combined stress uniquely remodels the hepatic metabolome.

The distribution of upregulated and downregulated DEMs is summarized in Figure 4C. Relative to the control, the S vs. C comparison yielded 136 upregulated and 174 downregulated metabolites; the L vs. C comparison showed 125 upregulated and 143 downregulated metabolites. In contrast, the S + L vs. C comparison identified 80 upregulated and 199 downregulated metabolites, with the number of downregulated metabolites being substantially higher than in either single-stress group. Moreover, in the direct comparison of combined stress versus cold alone (S + L vs. L), 86 upregulated and 215 downregulated metabolites were detected, further indicating a more pronounced metabolic suppression under dual stress. A heatmap of representative DEMs (Figure 4D) confirmed clear separation of the four groups and revealed distinct metabolite abundance patterns. The combined stress group exhibited characteristic changes in metabolites linked to lipid metabolism, amino acid metabolism, and oxidative stress responses, including oxidized lipids, acyl amino acids, and aromatic aldehydes—profiles that were distinct from those observed under single-stress conditions.

### 3.4. Functional Enrichment of Metabolic Pathways in Response to Cold and Starvation Stress

**Figure 5:**
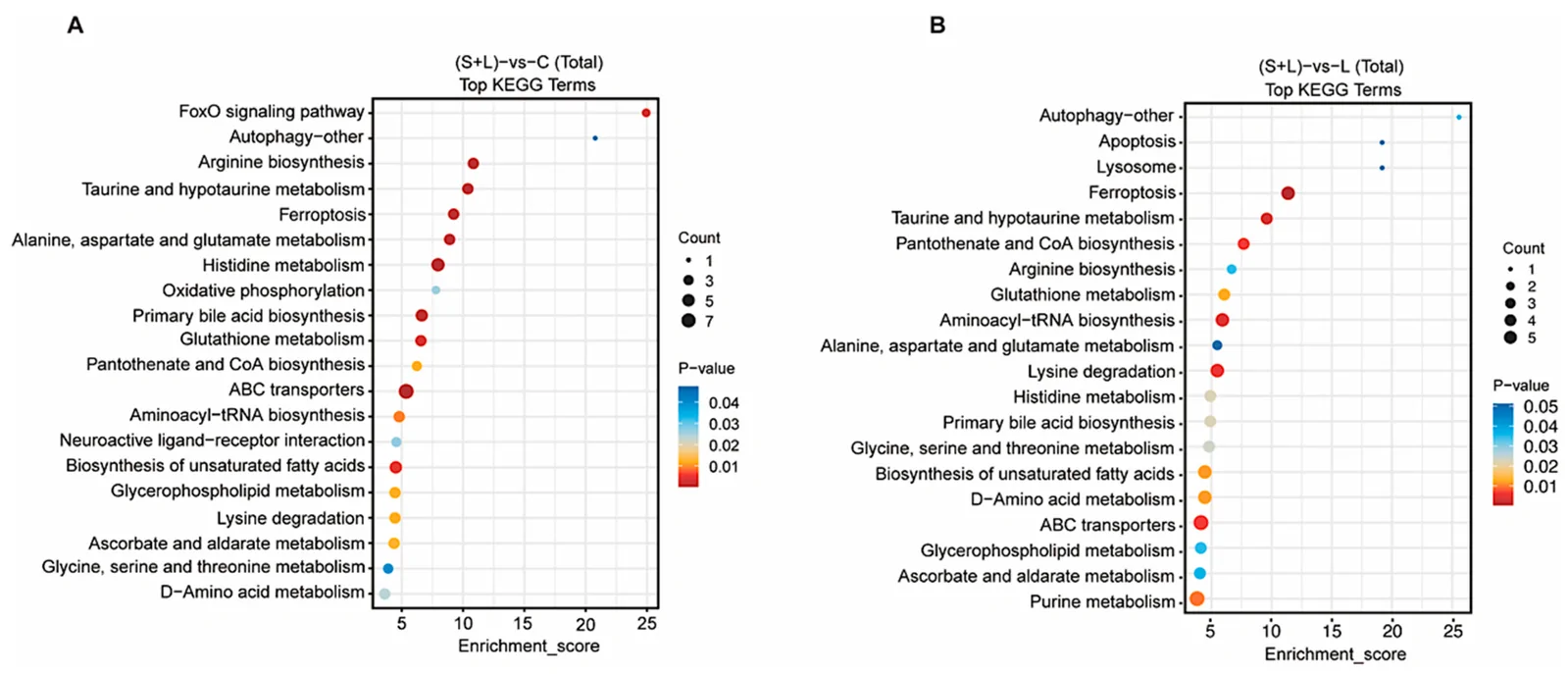
Functional enrichment analysis of differentially expressed metabolites (DEMs) in the liver of *P. vachelli* under combined cold and starvation stress (**A**) Top 20 KEGG enrichment pathways for DEMs in the combined stress group versus the control group. (**B**) Top 20 KEGG enrichment pathways for DEMs in the combined stress group versus the cold stress group.

To identify perturbed metabolic pathways under single and combined stress, KEGG enrichment was performed on differentially expressed metabolites (DEMs). Bubble plots summarize single-stress metabolic signatures (Figure S2); bar charts display pathway enrichment significance across four comparisons (Figure S3), and Figure S4 quantifies the up and downregulation proportions of pathways for cold-only and combined stress groups. In starvation-only fish (S vs. C, Figures S2A and S3A), amino acid, energy and glutathione metabolism, together with ABC transporters, constituted the dominant enriched pathways, with only mild ferroptosis enrichment, reflecting adaptive redox homeostasis without overt cell death activation under nutrient limitation. By contrast, cold-only stress (L vs. C, Figures S2B, S3B and S4A) triggered distinct metabolic remodeling: autophagy and FoxO signaling were enriched alongside lipid reprogramming, most amino acid and antioxidant pathways were suppressed, and ferroptosis exhibited mixed bidirectional metabolite regulation, indicating a mild stress response without severe metabolic collapse. Combined cold–starvation stress (S + L vs. C, Figure 5A and Figures S3C and S4B) induced hepatic metabolic collapse far more severe than either single stress. Although certain amino acid pathways (e.g., histidine metabolism, arginine biosynthesis, taurine and hypotaurine metabolism) remained moderately enriched, FoxO signaling, autophagy and ferroptosis achieved drastically elevated enrichment scores, representing hyperactivated cell death cascades. Directional quantification (Figure S4B) revealed prominent upregulation of oxidative phosphorylation; notably, glutathione metabolism–the core antioxidant defense pathway—was profoundly downregulated, alongside widespread repression of nearly all central amino acid, lipid and bile acid metabolic cascades. Further enrichment analysis of DEMs from S + L vs. L (Figure 5B and Figure S3D) identified core metabolic pathways and a highly significant cell death–lysosome functional module consisting of autophagy, apoptosis, lysosome, and ferroptosis pathways. Apoptosis, lysosome and ferroptosis displayed stronger enrichment than in the S + L vs. C contrast, verifying that superimposed starvation synergistically exacerbates lysosomal clearance and ferroptotic cell death beyond cold stress alone. Collectively, single starvation elicits adaptive amino acid and antioxidant metabolism, while cold stress represses basal amino acid turnover and moderately activates lipid catabolism and stress signaling. Only combined dual stress triggers systemic hepatic metabolic breakdown, characterized by drastically enriched ferroptosis, sharply upregulated oxidative phosphorylation (OXPHOS), robust suppression of glutathione-mediated antioxidant defense, and synchronous overactivation of FoxO, autophagy and apoptotic programs. This metabolic shift toward energy dissipation and unrestrained cell death signaling directly drives aggravated liver injury under co–occurring cold and starvation stress.

**Figure 6:**
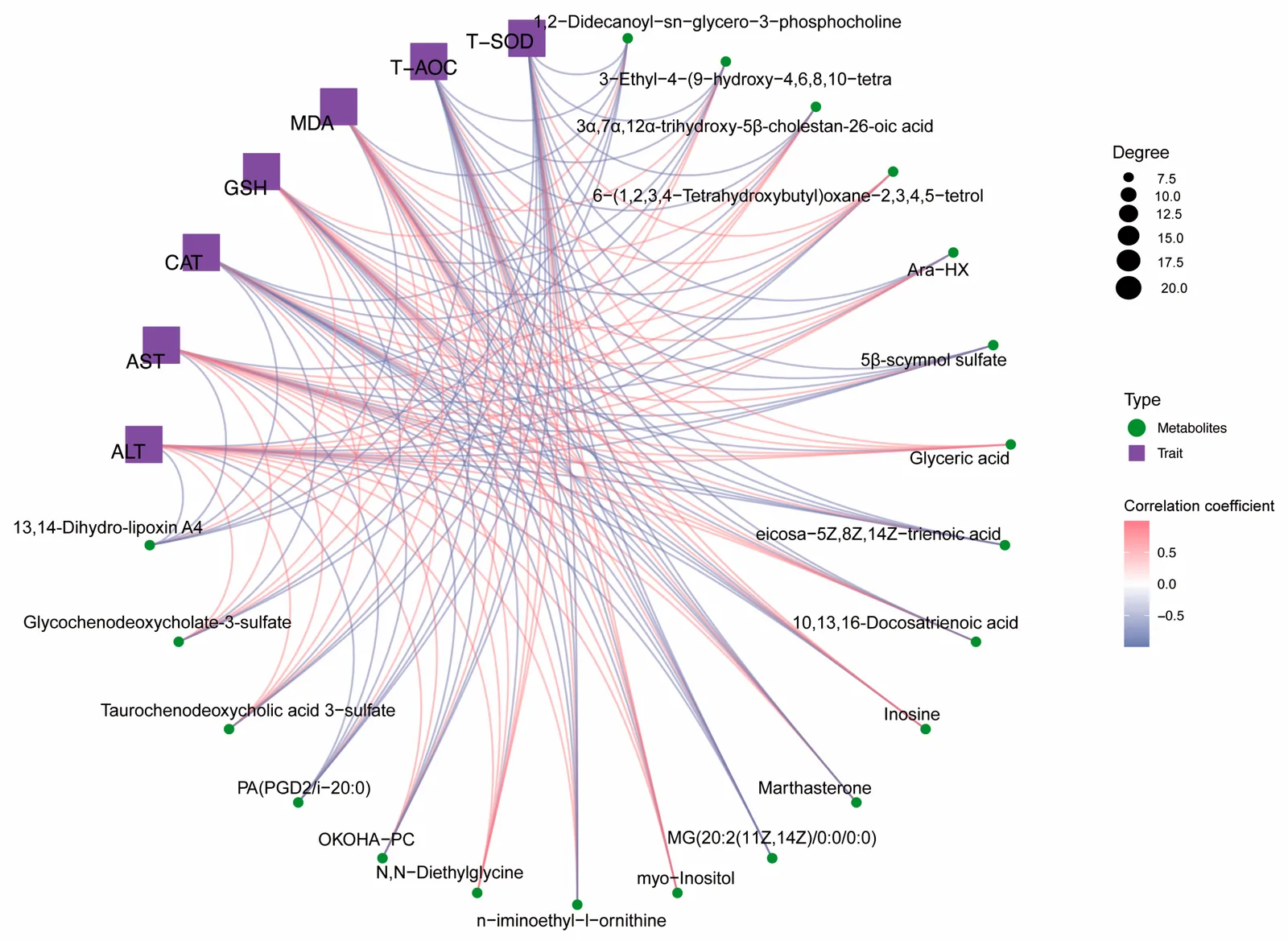
Integrative interaction network illustrating the associations between physiological indices and differentially expressed metabolites (DEMs). Edges indicate significant correlations (*p* < 0.05), with edge thickness proportional to correlation strength and edge color denoting positive (red) or negative (blue) associations.

To further explore the interplay between hepatic physiological dysfunction and metabolic reprogramming under combined cold and starvation stress, a correlation network integrating key liver biochemical indicators and significantly altered hepatic metabolites was constructed (Figure 6). The network revealed extensive correlations among liver injury markers (AST, ALT), oxidative stress indices (MDA, GSH, CAT, T–AOC, T–SOD), and metabolites. AST and ALT emerged as central hubs, showing strong positive correlations with bile acid derivatives (glycochenodeoxycholate 3 sulfate, taurochenodeoxycholic acid 3 sulfate), the energy-related intermediate glyceric acid, and the oxidized glycerophospholipid OKOHA PC, reflecting their association with liver injury severity. Metabolites involved in antioxidant defense exhibited distinct patterns: 13,14 dihydro lipoxin A4 and myo inositol were positively correlated with antioxidant indicators (GSH, CAT, T–AOC) and negatively correlated with MDA, suggesting a protective role against oxidative stress. Polyunsaturated fatty acids (e.g., eicosa 5Z,8Z,14Z trienoic acid) and inosine also showed characteristic correlations with stress-related parameters. Collectively, these results demonstrate that hepatic metabolic dysregulation is tightly coupled with liver injury and oxidative stress under combined stress. The coordinated changes in liver functional parameters and associated metabolites further confirm that dual stress drives profound metabolic reprogramming in the liver, which likely contributes to the progression of hepatic functional impairment.

### 3.5. Imbalance of Gut Microbiota in Response to Cold and Starvation Stress

**Figure 7:**
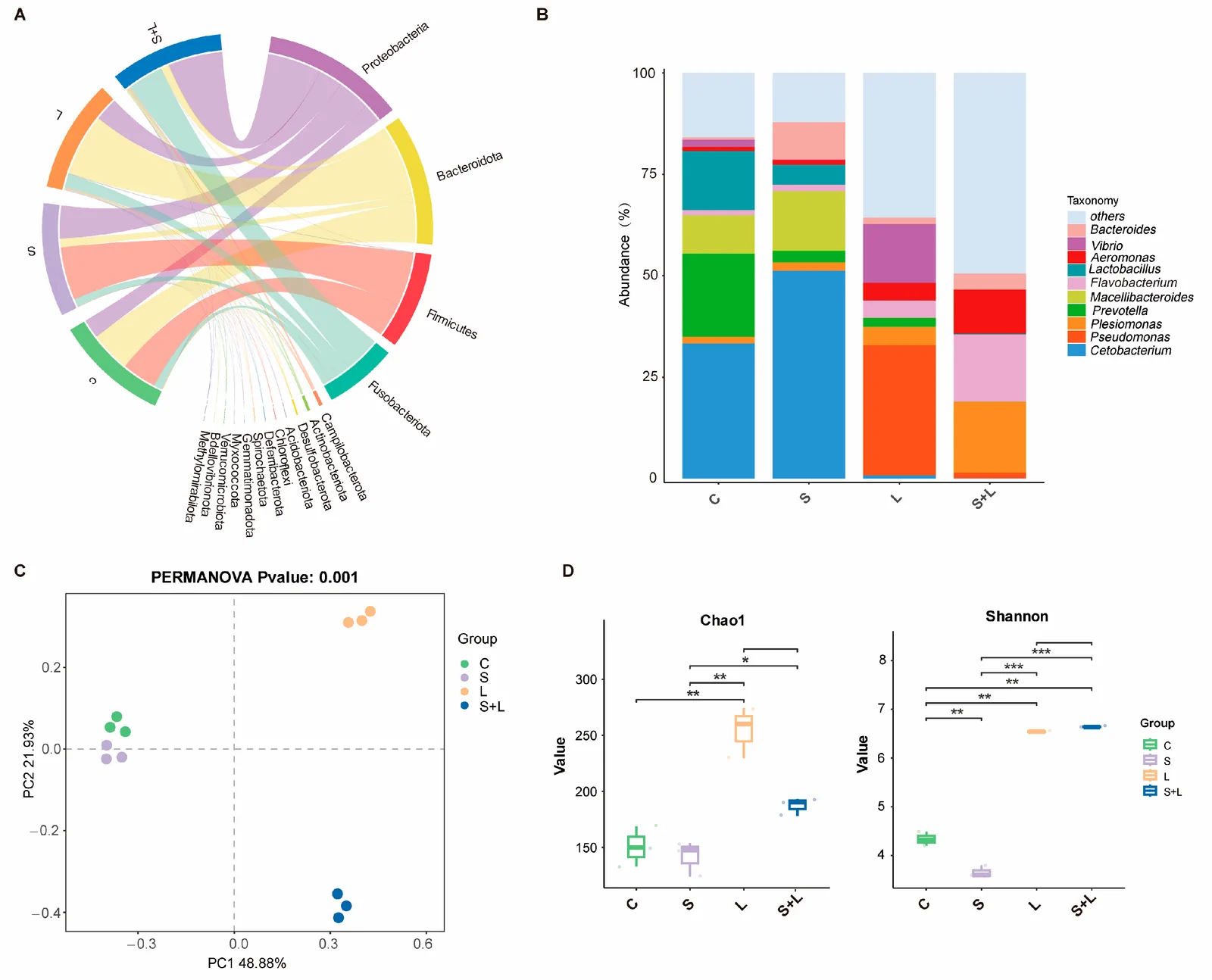
Composition structure and diversity analysis of the gut microbiota. (**A**) Microbial composition at the phylum level. (**B**) Microbial composition at the genus level. (**C**) PCA of microorganisms. (**D**) Chao1 and Shannon index. The control (C), starvation alone (S), cold alone (L), and combined (S + L) group. The asterisk represents a significant difference between the two groups. * *p* < 0.05, ** *p* < 0.01, *** *p* < 0.001.

To elucidate the effects of cold and starvation stress on gut microbial architecture, we systematically characterized the compositional shifts, diversity alterations, and key taxonomic variations in the intestinal bacterial community under these conditions. Figure 7A provides an overview of the phylum-level compositional landscape, revealing that Bacteroidota, Proteobacteria, Fusobacteriota, and Firmicutes were the dominant phyla across all experimental groups. At the genus level, the predominant taxa comprised *Cetobacterium*, *Plesiomonas*, *Pseudomonas*, *Flavobacterium* and *Lactobacillus* (Figure 7B). The abundance of *Cetobacterium* exhibited marked inter-treatment variation: it was highly prevalent in the control and starvation groups but declined sharply to nearly undetectable levels under combined cold and starvation stress. Conversely, the relative abundances of *Vibrio*, *Plesiomonas* and *Pseudomonas* were substantially elevated in the cold stress group, whereas *Plesiomona* and *Flavobacterium* emerged as the dominant genera in the S + L group (Figure 7B).

Beta diversity, assessed via principal coordinate analysis (PCA) based on Bray–Curtis distances, revealed significant dissimilarities in community structure among the four groups. Notably, samples from the S + L group formed a distinct cluster in the ordination space, clearly separated and far away from the other three groups, indicating that concurrent stress restructures the gut microbiota in a manner distinct from either single stressor alone (Figure 7C). Alpha diversity, evaluated using Chao1 and Shannon indices (Figure 7D), demonstrated that the Chao1 index (species richness) was significantly higher in the cold stress group relative to the control, whereas the Shannon index (community diversity) was markedly reduced in the starvation group. In contrast, the S + L group exhibited a significantly higher Shannon index than the C, S, and L groups, suggesting a unique community reorganization that could not be recapitulated by exposure to cold or starvation alone.

LEfSe analysis (LDA > 4) was further conducted to identify differentially abundant microbial biomarkers from the phylum to genus level (Figure S5). The cladogram distinctly illustrated discrepancies in microbial community structures across experimental groups. *Cetobacterium* and *Lactobacillus* were characterized as signature beneficial genera of the control group, while the S group was specifically enriched in Fusobacteriota–affiliated taxa, including *Cetobacterium* and *Epulopiscium*. Proteobacterial taxa, such as *Pseudomonas*, predominated in the cold stress group. Strikingly, the S + L group harbored a unique biomarker profile overwhelmingly composed of aquatic opportunistic pathogens, including *Plesiomonas*, *Pseudomonas*, and *Flavobacterium*. This pathogen-enriched signature was exclusive to the S + L group and corroborated the genus-level abundance patterns shown in Figure 7B, collectively confirming that concurrent cold and starvation stress selectively facilitates pathogenic bacterial colonization while suppressing beneficial commensals.

Collectively, cold and starvation stress individually altered gut microbial composition and diversity; however, their combination induced the most profound disruption of intestinal microbial homeostasis. Relative to single-stressor treatments, the S + L group exhibited a marked depletion of beneficial bacteria, robust proliferation of pathogenic taxa, and severe community dysbiosis, rendering its microbial profile clearly distinguishable from those of the other three experimental groups.

### 3.6. Functional Dysbiosis of Gut Microbiota Induced by Combined Cold and Starvation Stress

**Figure 8:**
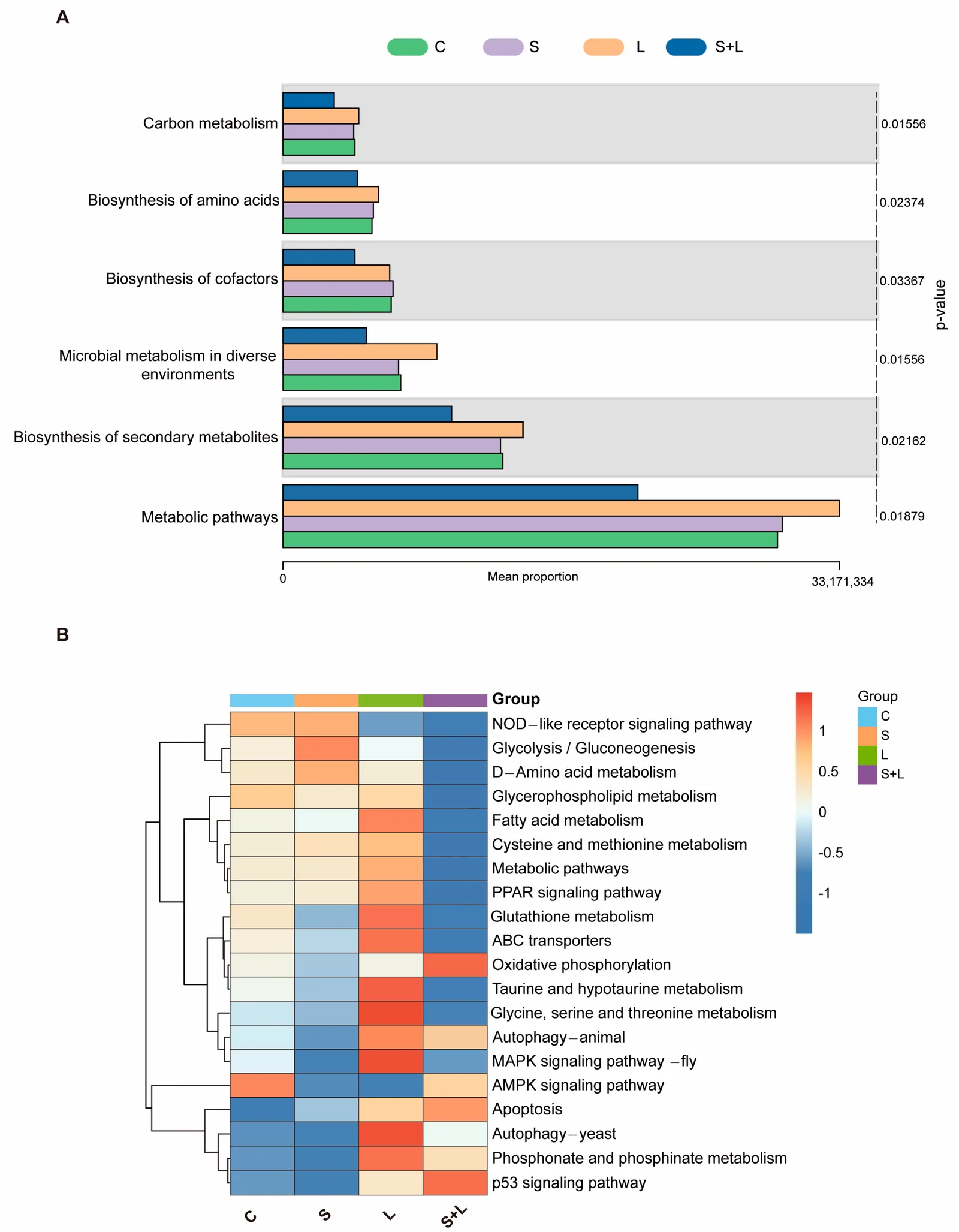
Microbiota function analysis of the KEGG pathways predicted by PICRUSt2. (**A**) Bar plot showing differentially abundant KEGG pathways at Level 2. (**B**) Clustered heatmap of relative abundance for KEGG pathways at Level 3. The control (C), starvation alone (S), cold alone (L), and combined (S + L) group.

To investigate functional alterations of the gut microbiota under different stress conditions, KEGG pathway-based functional prediction was performed (Figure 8). At KEGG level1, microbial functions were mainly classified into Metabolism, Environmental Information Processing, Cellular Processes, and Genetic Information Processing (Figure 8A). At KEGG level 2, core metabolic pathways (e.g., amino acid, carbohydrate, and energy metabolism) varied significantly among groups. Compared with the C group, the L and S + L groups showed higher relative abundances of these pathways, while the S group exhibited intermediate levels. A clustering heatmap of 20 representative KEGG level 3 pathways was generated (Figure 8B).

In the S group, only the glycolysis/gluconeogenesis pathway was significantly upregulated, while the vast majority of other pathways remained near control levels, indicating that short-term starvation induced the least functional perturbation of the gut microbiota among all stress treatments. In the L group, a broad spectrum of pathway reprogramming was observed: the immune-related NOD-like receptor pathway and the energy-sensing AMPK signaling pathway were significantly downregulated, whereas fatty acid metabolism, PPAR signaling, glutathione metabolism, ABC transporters, oxidative phosphorylation, taurine and hypotaurine metabolism, MAPK signaling, apoptosis, autophagy-animal, and p53 signaling pathways were all markedly upregulated. This pattern indicates that cold stress alone comprehensively activates microbial functional modules involved in lipid metabolism, antioxidant defense, and programmed cell death.

In the S + L group, compared with the control group, the NOD-like receptor, fatty acid metabolism, PPAR signaling, glutathione metabolism, ABC transporters, and taurine and hypotaurine metabolism pathways were significantly downregulated, whereas oxidative phosphorylation, apoptosis, autophagy, and p53 signaling pathways remained upregulated. Importantly, compared with the cold-alone group (L), the combined stress group exhibited further suppression of the NOD-like receptor immune pathway, suggesting that superimposed starvation exerts an additional inhibitory effect on microbial immune function. Collectively, single and combined stress treatments drove fundamentally different patterns of gut microbiota functional reprogramming. Short-term starvation specifically activated the glycolysis/gluconeogenesis pathway with minimal overall functional perturbation. Cold stress alone suppressed immune- and energy-sensing pathways while broadly activating lipid metabolism, antioxidant defense, and programmed cell death pathways. In contrast, combined cold and starvation stress deeply suppressed the NOD-like receptor immune pathway, reversed the cold-induced upregulation of fatty acid metabolism, PPAR signaling, and glutathione metabolism, yet retained the upregulation of oxidative phosphorylation, apoptosis, autophagy, and p53 signaling. These findings demonstrate that the functional landscape of gut microbiota under combined cold–starvation stress is qualitatively distinct from that induced by either single stressor alone.

### 3.7. Integrative Correlation Analysis Among Gut Microbiota, Hepatic Metabolome, and Liver Biochemical Indices Under Combined Cold and Starvation Stress

**Figure 9:**
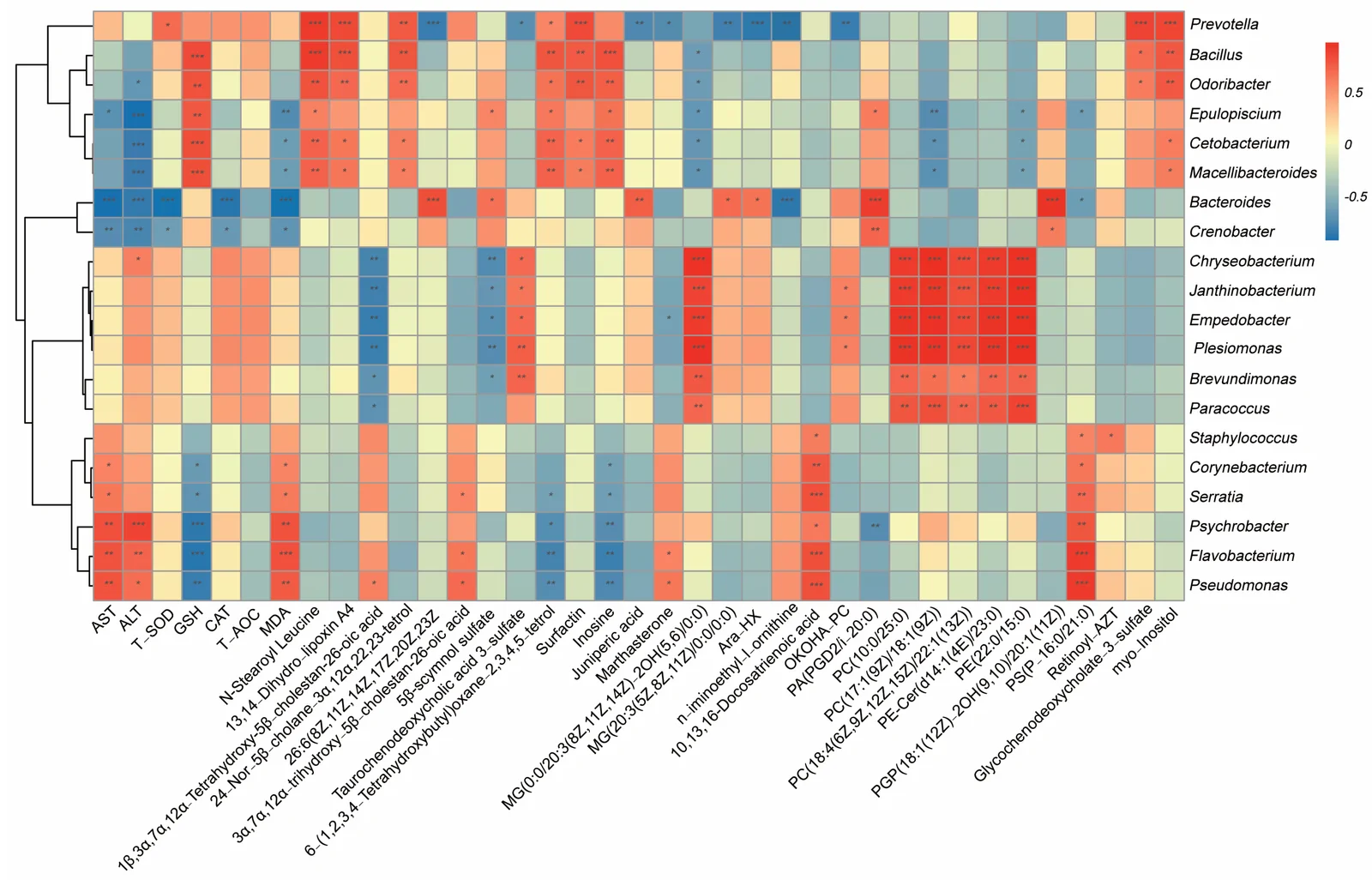
Spearman’s correlation analysis between gut microbiota at the genus level and physiological biomarkers as well as differentially expressed metabolites (DEMs) in liver tissue. Asterisks indicate significant differences (* *p* < 0.05, ** *p* < 0.01, ***, *p* < 0.001). Red and blue represent positive and negative correlations, respectively. Color intensity is proportional to the correlation magnitude.

To investigate the potential link among gut microbiota, hepatic function, and metabolic perturbations under combined cold and starvation stress, Spearman correlation analysis was performed between the gut bacterial genera, key hepatic biochemical indicators, and significantly altered hepatic metabolites (Figure 9). The heatmap revealed extensive correlations among gut microbiota, liver functional parameters, and metabolites. Several bacterial genera exhibited distinct correlation patterns with hepatic injury and oxidative stress markers. Putative pathogenic genera, including *Pseudomonas*, *Psychrobacter*, and Flavobacterium, were strongly positively correlated with liver injury indices (AST, ALT), whereas beneficial taxa such as *Macellibacteroides* and *Cetobacterium* showed negative correlations with these markers. Conversely, Bacteroides (a beneficial genus) was positively correlated with antioxidant indicators (GSH, CAT, T-AOC, T-SOD), while pathogenic genera like *Chryseobacterium* and *Janthinobacterium* displayed negative correlations with these antioxidant markers.

Notably, many significantly altered hepatic metabolites also showed characteristic correlations with specific gut bacteria. Bile acid derivatives (e.g., glycochenodeoxycholate-3-sulfate, taurochenodeoxycholic acid 3-sulfate), the oxidized glycerophospholipid OKOHA-PC, and the energy-related intermediate glyceric acid were strongly positively correlated with *Pseudomonas*, *Psychrobacter*, and *Flavobacterium*—genera that were also positively associated with AST and ALT. In contrast, these pathogenic genera were negatively correlated with polyunsaturated fatty acid derivatives and the anti-inflammatory metabolite 13,14-dihydro-lipoxin A4. Beneficial genera, including *Cetobacterium*, *Macellibacteroides*, and *Bacteroides*, displayed opposite correlation trends, being positively correlated with antioxidant/anti-inflammatory metabolites and negatively correlated with liver injury-related metabolites. Collectively, these correlation results demonstrate that gut microbiota are closely linked to both hepatic physiological status and metabolic profiles under combined stress.

**Figure 10:**
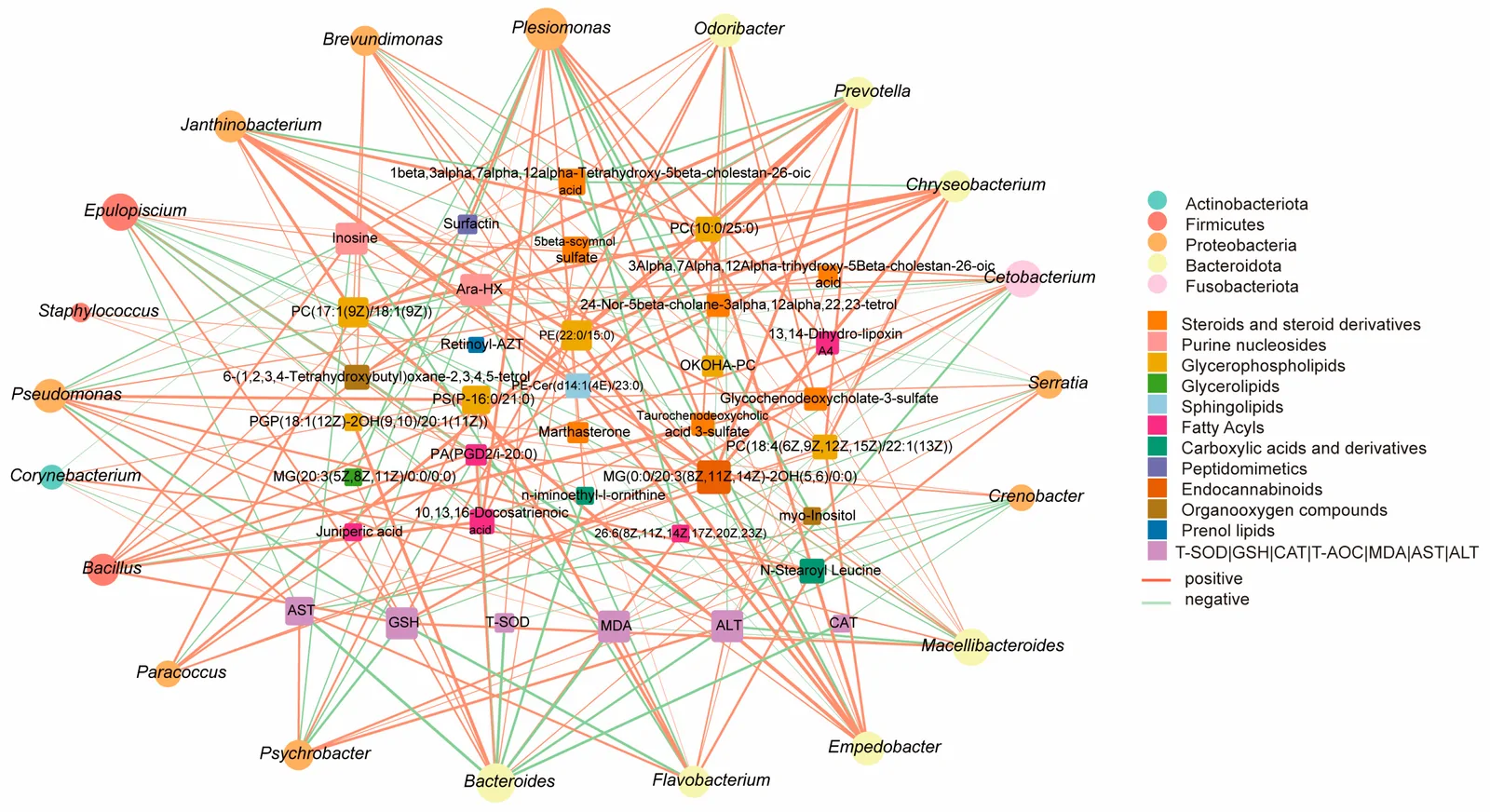
Integrative interaction network depicting the correlations among gut microbiota, physiological biomarkers, and differentially expressed metabolites (DEMs). Circles and squares represent bacterial genera and biomarkers/DEMs, respectively. Node color indicates phylum (microbiota) or metabolic pathway (metabolites); node size reflects abundance (genera) or centrality (biomarkers/metabolites). Edges denote significant correlations (*p* < 0.05), with thickness indicating strength, and green/red representing positive/negative associations.

Extending the pairwise correlation analyses described above, we next constructed a multi-omics network to systematically integrate gut bacterial genera, hepatic biochemical indicators, and differentially expressed metabolites under combined cold and starvation stress (Figure 10). This network allowed us to visualize the holistic interconnections among the three data layers. The network revealed a tightly interconnected landscape, wherein gut bacteria, liver injury markers (AST, ALT), oxidative stress indices, and metabolites formed distinct yet interlinked functional clusters. Notably, the most highly connected nodes in the network were several bacterial genera, including *Plesiomonas*, *Pseudomonas*, and *Flavobacterium*. These genera displayed extensive positive correlations with both liver injury indicators (AST, ALT) and a broad spectrum of hepatic damage-related metabolites–specifically, bile acid derivatives (glycochenodeoxycholate-3-sulfate, taurochenodeoxycholic acid 3-sulfate), the oxidized glycerophospholipid OKOHA-PC, and energy-related intermediates (e.g., glyceric acid). In striking contrast, beneficial taxa—Cetobacterium, Macellibacteroides, and Bacteroides—formed a distinct correlation module. They showed strong positive correlations with antioxidant indicators (GSH, CAT, T-AOC, T-SOD) and anti-inflammatory/cytoprotective metabolites, such as 13,14-dihydro-lipoxin A4 and myo-inositol, while being negatively associated with AST and ALT.

Collectively, this integrated network demonstrates that gut microbiota dysregulation is closely coupled with both hepatic dysfunction and metabolic reprogramming under combined stress. The coordinated tripartite correlations provide strong support for the hypothesis that gut–liver metabolic crosstalk critically mediates the host’s adaptive response to environmental stress.

## 4. Discussion

Concurrent low temperature and food deprivation represent a typical combined stressor driving massive mortality in farmed freshwater fish during overwintering. While extensive research has characterized physiological responses to either stress alone, and our previous work specifically characterized cold-induced hepatic injury in *P. vachelli* [35], systematic multi-omics dissection of gut–liver axis-mediated synergistic hepatic injury under coupled overwintering stress remains scarce. Critically, the hepatic phenotypes and metabolic reprogramming observed under dual stress are not merely additive aggravation of cold-induced damage; instead, superimposed starvation reshapes core metabolic networks and specifically amplifies ferroptotic cell death, revealing mechanistic features that cannot be predicted from single cold stress data. By integrating histopathology, serum biochemistry, 16S rRNA microbial profiling, and untargeted hepatic metabolomics in *P. vachelli* exposed to 11 °C cold and 10-day starvation, we demonstrate that combined stress triggers non-additive synergistic liver damage qualitatively distinct from single-stress responses. Two-way ANOVA confirmed significant interactive effects of cold and starvation on all hepatic injury biomarkers (*p* < 0.05), providing statistical evidence that starvation serves as a critical amplifier of cold–induced hepatic injury.

### 4.1. Starvation Amplifies Cold-Induced Hepatic Injury Through Energy Depletion

Hepatic histology revealed that combined cold and starvation stress drastically aggravated liver lesions. Compared with the L or S groups, the S + L group exhibited sharply elevated AST and ALT activities, massive accumulation of the lipid peroxidation product MDA, and significant downregulation of core antioxidant indicators including SOD, CAT, T-AOC, and GSH. Low temperature alone activates endogenous antioxidant compensatory programs in teleosts, as documented in hybrid *Larimichthys crocea* [39], *Eleutheronema tetradactylum* [40], and *Micropterus salmoides* [41]. Such compensatory machinery sustains basal hepatic homeostasis under short-term cold challenges. However, 10-day continuous starvation markedly suppressed these cold-triggered protective responses, a trend consistent with observations in co-stressed yellow drum (*Nibea albiflora*) [3].

The energy allocation paradigm explains this damage amplification: antioxidant defense and damaged organelle repair are highly energy-consuming processes. Sustained fasting depletes glycogen and lipid reserves, directly cutting off the energy supply for cold-induced compensatory responses, thereby converting reversible adaptive stress into persistent oxidative damage. Notably, short-term fasting (2–4 days) enhances cold resistance in *zebrafish* by stimulating lipolysis and autophagy [42,43,44]. In contrast, 10-day starvation in this study fully exhausted energy stores, shifting starvation from adaptive protection to pathological damage. Long-term dual-stress exposure induces growth retardation, profound hepatic metabolic disturbance, and irreversible hepatocellular vacuolar necrosis in several teleost species [45,46]. Nevertheless, the severity and timing of these injuries vary markedly across different fish species. Collectively, once stress duration exceeds the energy storage threshold, cold and starvation synergistically induce organic hepatic lesions, accounting for the phenotypic divergence between laboratory single-cold trials and field overwintering damage.

### 4.2. Metabolic Reprogramming Shifts from Glutathione Depletion to Energy Collapse and Ferroptosis Perturbation

Metabolic plasticity is a core adaptive strategy for fish coping with environmental stress. Metabolomic data revealed that combined stress reshaped a hepatic metabolic landscape entirely distinct from single-stress profiles, with perturbations in three core pathways collectively driving synergistic liver injury. Glutathione metabolism suppression intensified with increasing stress complexity. GSH is an essential cofactor for GPX4, which eliminates lipid peroxides and inhibits ferroptosis [47,48]. Massive GSH consumption under combined stress implies impaired GPX4 activity, raising hepatocyte susceptibility to ferroptosis. Glutamate and GSSG disturbances in dual-stressed yellow drum and large yellow croaker validate glutathione metabolism as a shared biomarker for fish responding to coupled stress [23,49].

Oxidative phosphorylation (OXPHOS)-associated metabolites were uniquely enriched only in the S + L group, with no comparable signature in either single-stress treatment. We hypothesize that starvation triggers compensatory mitochondrial respiration to offset energy deficits, yet aberrant OXPHOS activation continuously releases mitochondrial ROS, exacerbating lipid peroxidation and forming a vicious oxidative cycle [50,51]. This signature matches OXPHOS enrichment in co-stressed large yellow croaker [23]. A cold-stress trial on closely related yellow catfish also reported OXPHOS disorder [52], which may imply starvation as a prerequisite for mitochondrial dysfunction, with cold further amplifying oxidative injury. KEGG enrichment further revealed superimposed perturbation of multiple pathways under combined stress.

Metabolites linked to FoxO signaling and autophagy remained persistently enriched in the S + L group, while ferroptosis, taurine/hypotaurine metabolism, and lysine degradation exhibited far stronger disturbance than in the L group. Autophagy exerts dual effects during stress responses: moderate activation clears damaged organelles to confer protection, yet prolonged perturbation synergistically governs multiple programmed cell death pathways [42]. Comparative analysis between S + L and L groups demonstrated that starvation specifically exacerbates ferroptosis-linked metabolic disorders, transforming metabolically plastic livers under cold exposure into pathological states with catabolic imbalance and simultaneous activation of multiple cell death cascades.

### 4.3. Starvation Drives Irreversible Gut Microbial Dysbiosis and Ferroptosis Perturbation

Stable intestinal microbiota serves as a vital physiological barrier against cold-triggered oxidative damage. 16S rRNA sequencing revealed that combined cold–starvation stress induced radical dysbiosis: core beneficial genera *Cetobacterium* and *Bacteroides* declined sharply, while opportunistic pathogens *Plesiomonas*, *Pseudomonas*, and *Flavobacterium* underwent explosive proliferation. Progressive enrichment of Pseudomonas and Flavobacterium under cold acclimation has been documented in silver pomfret (*Pampus argenteus*) [53], olive flounder (*Paralichthys olivaceus*) [54], and our previous single-cold trial on *P. vachelli* [35]. As typical aquaculture opportunistic pathogens, they proliferate rapidly when host antioxidant and immune defenses decline, disrupting mucosal barriers and releasing pro-inflammatory metabolites.

Notably, *Plesiomonas* expansion occurred synchronously with hepatic ferroptosis perturbation in the S + L group. Pathogen challenge assays confirmed that *Plesiomonas* shigelloides infection simultaneously induces ferroptosis and apoptosis in zig-zag eel (*Mastacembelus armatus*) hepatopancreas [55]. Elevated intestinal *Plesiomonas* was also detected in cold-exposed yellow catfish (*Pelteobagrus fulvidraco*) and *P. vachelli* [35,52]. We hypothesize that excessive intestinal *Plesiomonas* may participate in hepatocellular ferroptosis regulation, though causal relationships await validation via microbiota transplantation and gene interference assays. Sustained beneficial microbe depletion impairs SCFA-mediated anti-inflammatory signaling and intestinal epithelial barrier integrity, establishing a self-reinforcing cascade, wherein intestinal barrier disruption permits translocation of toxic metabolites via the portal vein, which in turn triggers hepatic metabolic dysregulation and ultimately drives irreversible organic injury [56,57]. Integrating hepatic metabolic signatures further refines this cascade: severe GSH depletion compromises anti-ferroptosis defenses, while aberrant OXPHOS continuously generates lipid peroxidation substrates, collectively creating a microenvironment permissive to ferroptosis. Meanwhile, massive pathogen proliferation intensifies lipid peroxidation and intracellular iron accumulation, jointly elevating programmed cell death risk. Microbial dysbiosis and hepatic metabolic imbalance form a bidirectional gut–liver axis network, reciprocally amplifying one another and driving irreversible progression of synergistic hepatic damage.

### 4.4. Multi-Omics Correlation Network Uncovers Regulatory Patterns of Gut–Liver Axis-Mediated Synergistic Injury

To systematically integrate microbial taxa, hepatic metabolites, and physiological biomarkers, a correlation network was constructed. Network analysis revealed a highly interconnected interactome linking gut microbiota, hepatic metabolites, and biochemical indicators. Pathogenic genera (*Plesiomonas*, *Pseudomonas*, *Flavobacterium*) constituted core injury hubs, exhibiting significant positive correlations with hepatotoxic bile acid derivatives, oxidized glycerophospholipid OKOHA-PC, and hepatic leakage markers AST/ALT. In contrast, beneficial genera (*Cetobacterium*, *Bacteroides*) clustered with antioxidant biomarkers (T–SOD, GSH, CAT, T–AOC) and the anti-inflammatory metabolite 13,14-dihydro-lipoxin A4, forming a protective module. This bipartite structure demonstrates that microbe–host crosstalk under combined stress is non-random, segregating into distinct pro-inflammatory and protective response axes.

Multi-omics correlation networks are a mainstream framework for deciphering host–microbe interactions under environmental stress. Integrated microbiome and metabolome profiling has constructed gut–liver axis toxicity transmission networks in fish exposed to microplastics, contaminants and heavy metals [29,58,59]. Cold-stress trials on common carp (*Cyprinus carpio*) also reported strong correlations between cold-induced intestinal dysbiosis and hepatic metabolic disturbance [31]. These studies collectively validate that multi-dimensional correlation networks efficiently decode coordinated microbe–metabolite–liver responses.

**Figure 11:**
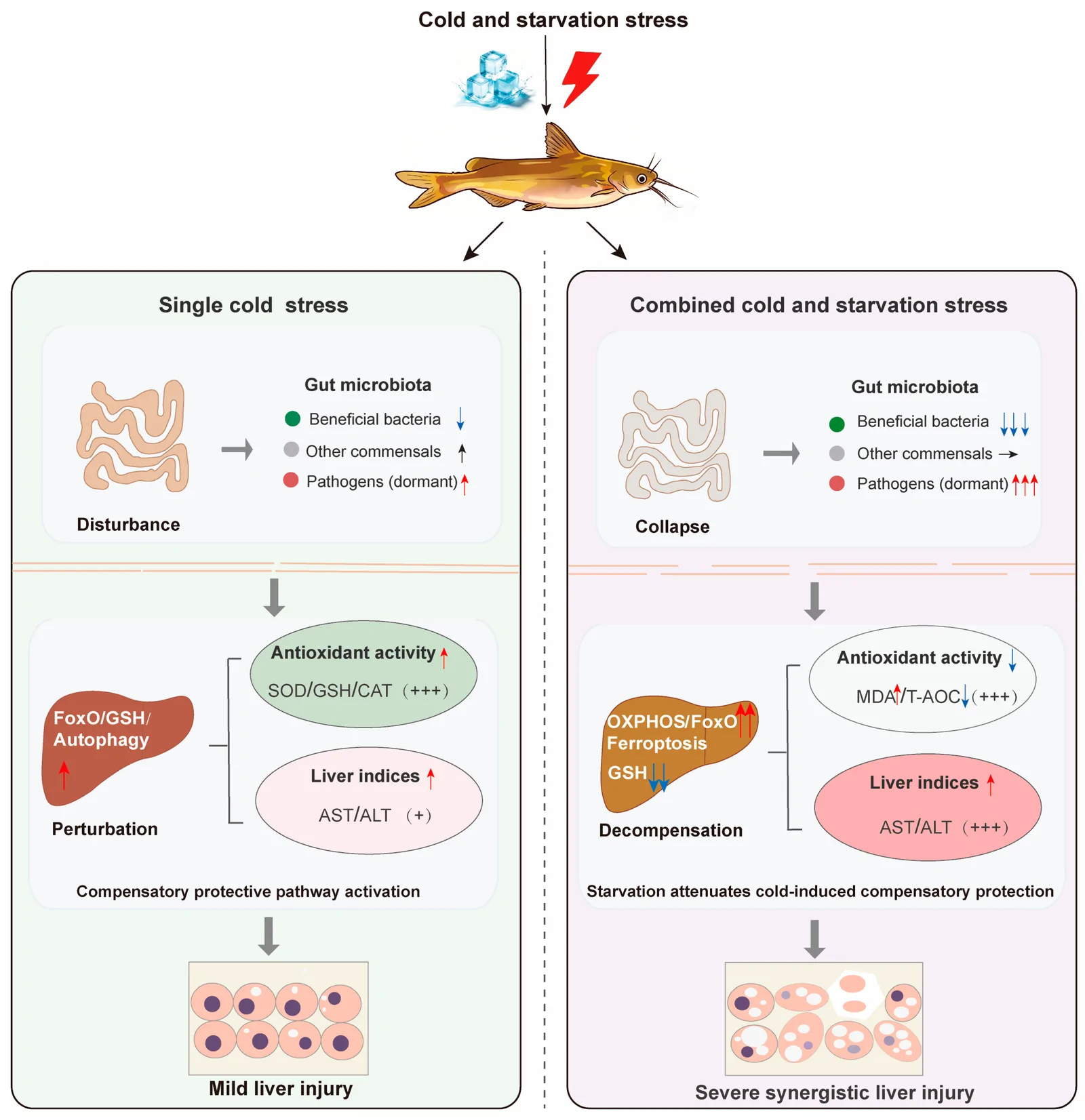
Proposed mechanism model of synergistic liver injury induced by combined cold and starvation stress via gut–liver axis disruption in *P. vachelli*. (**Left**) panel (single cold stress, 11 °C, feeding): Moderate hepatic injury with mild vacuolation, moderately elevated AST/ALT/MDA, partial induction of SOD, GSH and CAT, and compensatory moderate autophagy and apoptosis. The gut exhibits mild dysbiosis: moderately reduced beneficial *Cetobacterium* and slightly expanded opportunistic *Plesiomonas*. (**Right**) panel (combined cold–starvation stress, 11 °C, fasting): Severe synergistic hepatic injury with aggravated necrosis and vacuolation, drastically increased AST/ALT/MDA and suppressed antioxidant enzyme activity. Starvation eliminates cold-triggered hepatic protection and weakens stress resilience. Unique metabolic reprogramming co-activates FoxO, ferroptosis, autophagy, apoptosis and oxidative phosphorylation, driving glutathione depletion. Severe gut dysbiosis arises, with accumulated pathogens (*Plesiomonas*, *Pseudomonas*, *Flavobacterium*) and depleted probiotic taxa (*Cetobacterium*, *Lactobacillus*). Severe gut–liver axis breakdown facilitates bacterial translocation, exacerbating hepatic oxidative damage and inflammation; pathogenic bacteria act as core hubs connecting intestinal dysbiosis to hepatic metabolic dysfunction. Color code: red arrows: activation/aggravation; blue arrows: inhibition; dashed lines: bacterial translocation; white circles: vacuolization; purple circles: cell nuclei; pink circles: hepatocytes.

The dual destructive effects of starvation on both terminals of the gut–liver axis are reflected through this network. At the intestinal level, starvation disrupts commensal communities and removes inhibitory constraints on pathogen proliferation. At the hepatic level, starvation suppresses GSH synthesis and disturbs mitochondrial energy metabolism, lowering hepatic tolerance to microbe-derived toxins. Concurrent breakdown of intestinal barrier and hepatic defense may contribute to injury amplification through bidirectional gut–liver axis signaling, ultimately aggravating synergistic liver damage. Notably, these network-based associations are correlational rather than causal; functional validation studies (e.g., microbiota transplantation, gene knockdown) are required to establish direct mechanistic relationships. Based on the multi-omics findings described above, a schematic model is proposed to systematically illustrate the hypothesized synergistic liver injury mechanism induced by combined cold and starvation stress via gut–liver axis disruption (Figure 11).

### 4.5. Ecological Implications and Practical Management Strategies

This study clearly distinguishes the fundamental divergence between laboratory single cold stress and coupled cold–starvation stress under natural overwintering conditions. In field scenarios, cold and food scarcity co-occur, with starvation converting mild reversible cold–induced lesions into severe irreversible hepatic damage. The time–window effect, in which short–term fasting confers cold tolerance [43], while prolonged starvation triggers metabolic collapse, accounts for the divergent survival rates observed in overwintering populations [14]. Field surveys on large yellow croaker confirmed that starvation’s regulatory effects exhibit obvious time dependence [60].

Based on these findings, two practical management strategies are proposed. Complete feed deprivation should be avoided during cold snaps, and basic feeding rations should be maintained. Moderate exogenous nutrient supply preserves bodily energy reserves, stabilizes beneficial intestinal microbes, sustains hepatic GSH synthesis and antioxidant capacity, and blocks persistent oxidative stress and ferroptosis cascades. Research on common carp overwintering gut–liver axis verified that sustained moderate feeding alleviates cold-induced intestinal barrier breakdown and hepatic metabolic disorder [31].

Additionally, targeted microecological modulators (probiotics, prebiotics) adapted to low-temperature overwintering can be developed, targeting pathogenic genera (*Plesiomonas*, *Pseudomonas*, *Flavobacterium*) and beneficial taxa (*Cetobacterium*, *Bacteroides*). *Bacillus coagulans* BC66 has been shown to remodel intestinal microbiota of overwintering grass carp (*Ctenopharyngodon idella*), balance the microbe–metabolite–immune network, and markedly reduce overwintering syndrome mortality [61]. Analogous strategies can be translated to overwintering health management of *P. vachelli*, mitigating cascaded gut–liver axis injury triggered by combined cold and starvation. It should be noted that these proposed management strategies are inferred from laboratory stress experiments and require further dedicated feeding trials to validate their efficacy in practical aquaculture.

## 5. Conclusions

Integrated multi-omics evidence confirms that combined cold and starvation stress induces non-additive synergistic hepatic injury in *P. vachelli* via gut–liver axis disruption, as supported by the significant cold–starvation interaction observed in two-way ANOVA (*p* < 0.05). Three core findings emerge. First, starvation suppresses cold-activated antioxidant compensation through energy depletion, activating the OXPHOS–glutathione depletion–ferroptosis cascade. Second, starvation drives intestinal microbiota from mild fluctuation to irreversible collapse, with *Plesiomonas*, *Pseudomonas*, and *Flavobacterium* as core pathogenic hubs; *Plesiomonas* expansion is tightly coupled with hepatic ferroptosis. Third, starvation dismantles protective barriers at both ends of the gut–liver axis, establishing a self-amplifying cycle—intestinal leakage, metabolite translocation, metabolic disorder, and ferroptosis that synergistically amplifies liver damage.

The L vs. S + L comparison in this laboratory-based simulation demonstrates that starvation converts moderate reversible cold injury into severe irreversible damage. These findings, together with the time-dependent nature of starvation effects reported previously, advance the theoretical framework of cold stress and overwintering adaptation in *P. vachelli*, and provide practical references for feeding management and microecological regulation in aquaculture. Future optimization studies, including dose–response feeding trials, probiotic efficacy tests, and water environment regulation research, are warranted to mitigate cold-starvation stress and improve overwintering health management in aquaculture.

## Data Availability

The datasets presented in this article are not readily available because the raw datasets include materials relevant to other ongoing unpublished research. Requests to access the datasets should be directed to the corresponding authors.

## Supplementary Materials

The following supporting information can be downloaded at: https://www.mdpi.com/article/10.3390/antiox15080962/s1, Figure S1: Interactive effects of temperature and feeding on hepatic ALT (A) and AST (B) activities. Two-way ANOVA revealed significant cold–starvation interactions for both enzymes (*p* < 0.05). Under cold stress (11 °C), starvation significantly elevated ALT and AST versus feeding (*p* < 0.05). Data are mean ± SEM (n = 3 replicate tanks). Figure S2: Functional enrichment analysis of differentially expressed metabolites (DEMs) in the liver of *P. vachelli* under combined cold and starvation stress. (A) Top 20 KEGG enrichment pathways for DEMs in the S group versus the C group. (B) Top 20 KEGG enrichment pathways for DEMs in the L group versus the C group. Figure S3: Functional enrichment analysis of differentially expressed metabolites (DEMs) in the liver of *P. vachelli* under cold and/or starvation stress. (A) KEGG enrichment analysis of DEMs in the S group versus the C group. (B) KEGG enrichment analysis of DEMs in the L group versus the C group. (C) KEGG enrichment analysis of DEMs in the S + L group versus the C group. (D) KEGG enrichment analysis of DEMs in the S + L group versus the L group. Figure S4: Comparative visualization of the top 10 KEGG-enriched metabolic pathways associated with up- and downregulated differentially expressed metabolites (DEMs). (A) The L group versus C group. (B) The S + L group versus C group. Figure S5: Taxonomic distribution and differential abundance of gut microbiota in *P. vachelli* under cold and starvation stress. Histogram of the LDA scores, where the LDA score indicates the effective size and ranking of each differentially abundant taxon. The taxa with Kruskal—Wallis < 0.05 and LDA > 4 are shown.
