# Supplementary material for: Starvation Exacerbates Cold-Induced Synergistic Hepatic Injury in Pelteobagrus vachelli via Gut–Liver Axis Disruption and Ferroptosis-Related Metabolic Reprogramming
Source: Antioxidants (Basel). 2026 Jul 31;15(8):962. doi: 10.3390/antiox15080962 (PMC13510165; doi:10.3390/antiox15080962)
Supplement: Supplementary file 1 [file antioxidants-15-00962-s001.zip › antioxidants-4451257-supplementary.pdf]

Supplementary materials

Starvation Exacerbates Cold-Induced Synergistic Hepatic Injury in Pelteobagrus vachelli via Gut–Liver Axis Disruption and Ferroptosis–Related Metabolic Reprogramming

Amei Liu[1,2,3*], Libo Yang[4], Yuting Hu[1,2,3], Huaxing Zhou[1,2,3], Huan Wang[1,2,3], Guoqing Duan[1,2,3*]

1. Institute of Fisheries Science, Anhui Academy of Agricultural Sciences, Nongke South Road, Hefei 230031, China
2. Anhui Province Key Laboratory of Aquaculture & Stock Enhancement, Hefei 230031, China
3. Anhui Agricultural Germplasm Resource Center, Anhui Academy of Agricultural Sciences, Hefei 230031, China
4. Institute of Artificial Intelligence, Hefei Comprehensive National Science Center, Hefei 230088, China

* Correspondence: duanguoqing@aaas.org.cn (G. D.); liuamei@aaas.org.cn (A.L.)

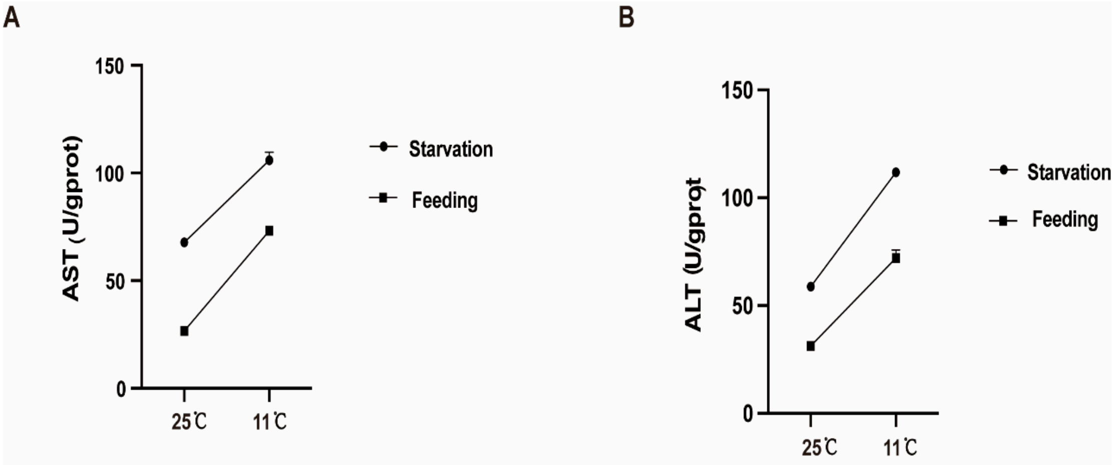

Supplemental Figure S1. Interactive effects of temperature and feeding status on hepatic ALT (A) and AST (B) activities. Two-way ANOVA revealed significant cold–starvation interactions for both enzymes ($P < 0.05$). Under cold stress (11 °C), starvation significantly elevated ALT and AST versus feeding ($P < 0.05$). Data are mean ± SEM ($n = 3$ replicate tanks).

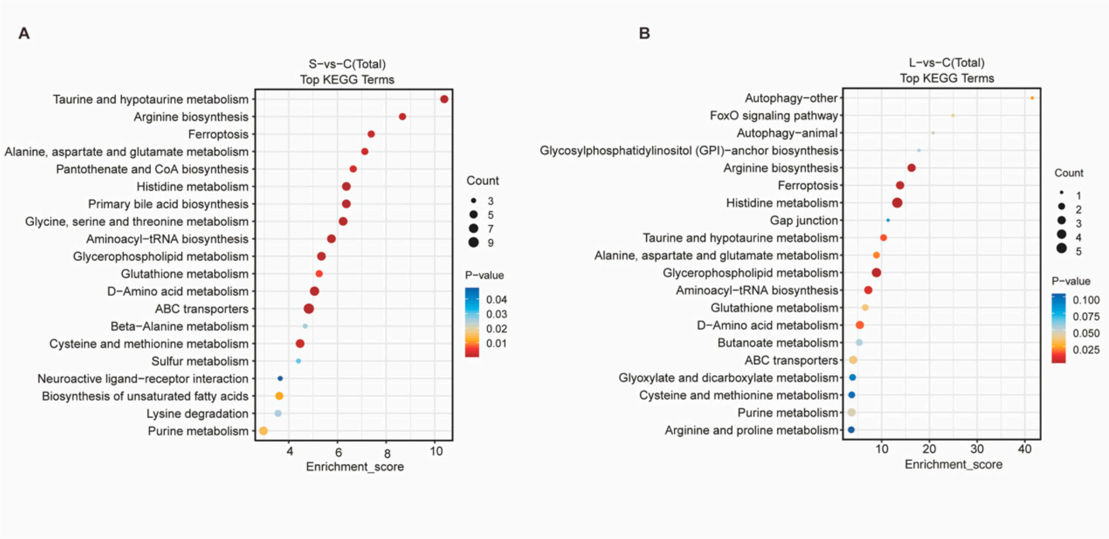

Supplemental Figure S2. Functional enrichment analysis of differentially expressed metabolites (DEMs) in the liver of *P. vachelli* under combined cold and starvation stress. (A) Top 20 KEGG enrichment pathways for DEMs in the S group versus the C group. (B) Top 20 KEGG enrichment pathways for DEMs in the L group versus the C group.

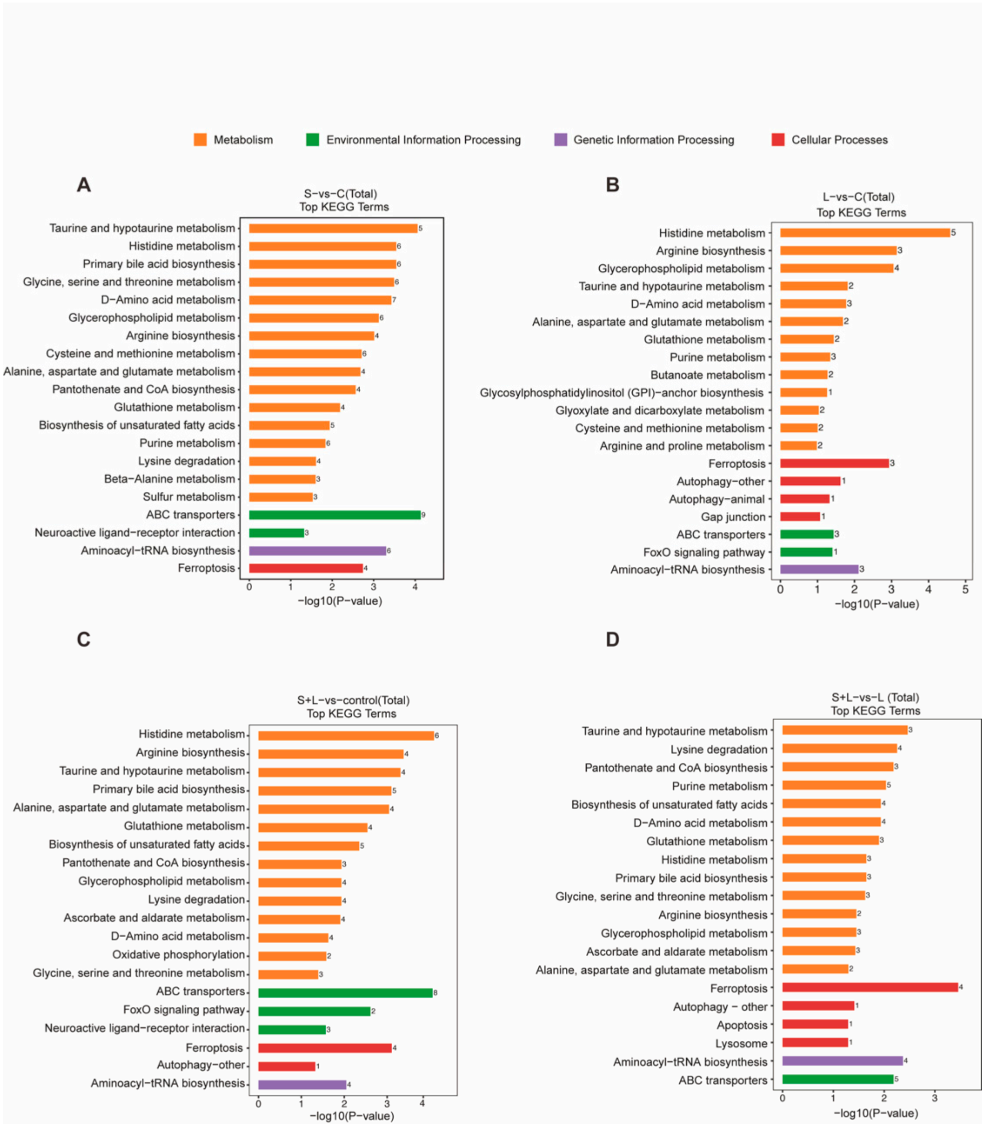

Supplemental Figure S3. Functional enrichment analysis of differentially expressed metabolites (DEMs) in the liver of *P. vachelli* under cold and/or starvation stress. (A) KEGG enrichment analysis of DEMs in the S group versus the C group. (B) KEGG enrichment analysis of DEMs in the L group versus the C group. (C) KEGG enrichment analysis of DEMs in the S+L group versus the C group. (D) KEGG enrichment analysis of DEMs in the S+L group versus the L group.

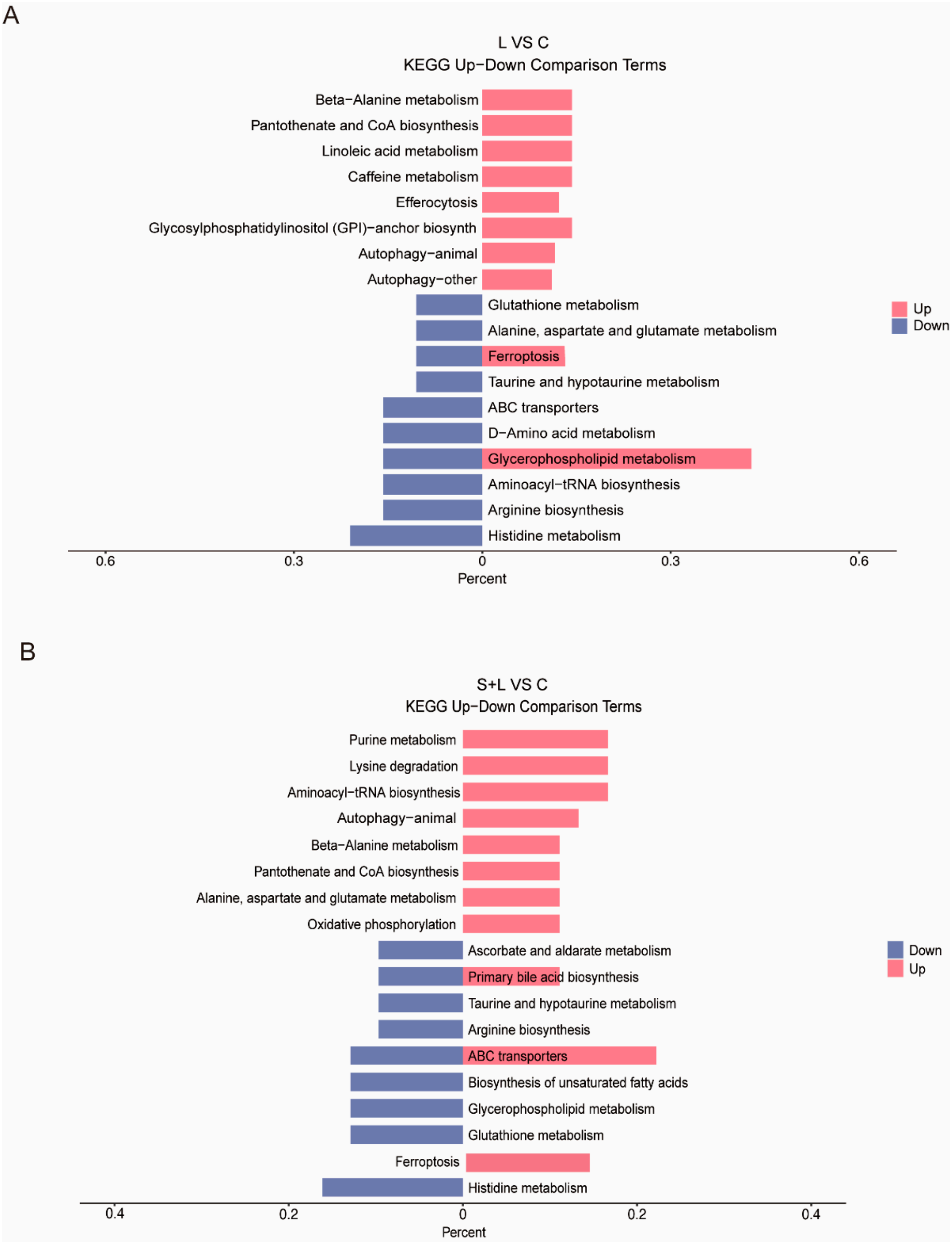

Supplemental Figure S4. Comparative visualization of the top 10 KEGG－enriched metabolic pathways associated with up-and downregulated differentially expressed metabolites (DEMs). (A) The L group versus the C group. (B) The S+L group versus the C group.

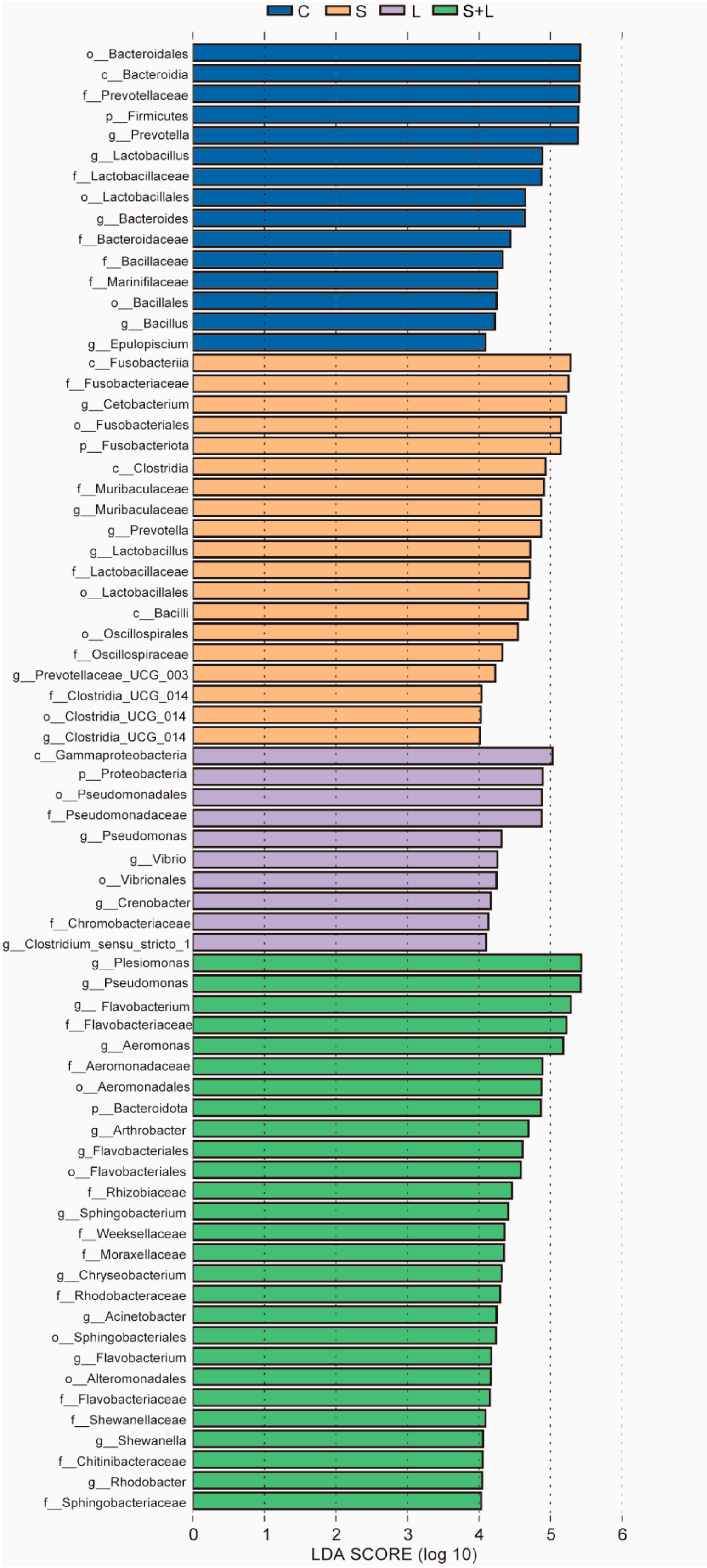

Supplemental Figure S5. Taxonomic distribution and differential abundance of gut microbiota in *P. vachelli* under cold and starvation stress. Histogram of the LDA scores, where the LDA score

indicates the effective size and ranking of each differentially abundant taxon. The taxa with Kruskal—Wallis < 0.05 and LDA > 4 are shown.
